# Platelet Gene Therapy Promotes Targeted Peripheral Tolerance by Clonal Deletion and Induction of Antigen-Specific Regulatory T Cells

**DOI:** 10.3389/fimmu.2018.01950

**Published:** 2018-09-06

**Authors:** Xiaofeng Luo, Juan Chen, Jocelyn A. Schroeder, Kenneth P. Allen, Christina K. Baumgartner, Subramaniam Malarkannan, Jianda Hu, Calvin B. Williams, Qizhen Shi

**Affiliations:** ^1^Blood Research Institute, BloodCenter of Wisconsin, Milwaukee, WI, United States; ^2^Fujian Institute of Hematology, Fujian Provincial Key Laboratory on Hematology, Fujian Medical University Union Hospital, Fuzhou, China; ^3^Departments of Pediatrics, Medicine, Microbiology and Immunology, and Biomedical Resource Center, Medical College of Wisconsin, Milwaukee, WI, United States; ^4^Children's Research Institute, Children's Hospital of Wisconsin, Milwaukee, WI, United States; ^5^MACC Fund Research Center, Milwaukee, WI, United States

**Keywords:** immune tolerance, platelet, gene therapy, clonal deletion, Treg induction

## Abstract

Delivery of gene therapy as well as of biologic therapeutics is often hampered by the immune response of the subject receiving the therapy. We have reported that effective gene therapy for hemophilia utilizing platelets as a delivery vehicle engenders profound tolerance to the therapeutic product. In this study, we investigated whether this strategy can be applied to induce immune tolerance to a non-coagulant protein and explored the fundamental mechanism of immune tolerance induced by platelet-targeted gene delivery. We used ovalbumin (OVA) as a surrogate non-coagulant protein and constructed a lentiviral vector in which OVA is driven by the platelet-specific αIIb promoter. Platelet-specific OVA expression was introduced by bone marrow transduction and transplantation. Greater than 95% of OVA was stored in platelet α-granules. Control mice immunized with OVA generated OVA-specific IgG antibodies; however, mice expressing OVA in platelets did not. Furthermore, OVA expression in platelets was sufficient to prevent the rejection of skin grafts from CAG-OVA mice, demonstrating that immune tolerance developed in platelet-specific OVA-transduced recipients. To assess the mechanism(s) involved in this tolerance we used OTII mice that express CD4^+^ effector T cells specific for an OVA-derived peptide. After platelet-specific OVA gene transfer, these mice showed normal thymic maturation of the T cells ruling against central tolerance. In the periphery, tolerance involved elimination of OVA-specific CD4^+^ effector T cells by apoptosis and expansion of an OVA-specific regulatory T cell population. These experiments reveal the existence of natural peripheral tolerance processes to platelet granule contents which can be co-opted to deliver therapeutically important products.

## Introduction

Immune responses to transgene products or viral vectors are a major concern during gene therapy (1–4). Immune responses are also implicated in decreasing the therapeutic value of many biologic drugs (5–7). An example of the latter is the use of factor VIII (FVIII) or factor IX (FIX) coagulant protein replacement therapy for patients with hemophilia A or hemophilia B, respectively. Introducing a therapeutic protein via gene therapy is an attractive alternative for hemophilia treatment. However, immune response can occur to FVIII or FIX or to the viral vector during such approaches (8–14). To prevent adverse immune responses that target transduced cells and neo-proteins, immune modulation strategies using immunosuppressive medications have been explored in animal models (13, 15–24). An ideal gene therapy for hemophilia will deliver the missing protein at sustained levels and promote immune tolerance.

While expression of FVIII in hematopoietic cells has recently shown promise for treating hemophilia A in animal models, immune responses remain a significant concern (8, 25–30). Our previous studies have demonstrated that platelet-targeted expression of FVIII or FIX under control of the platelet-specific αIIb promoter (2bF8 or 2bF9) via lentiviral gene delivery to hematopoietic stem cells (HSCs) can efficiently rescue the hemophilic bleeding phenotype and induce antigen-specific immune tolerance in hemophilia mouse models (31–33). Our further studies have shown that FVIII-specific immune tolerance in hemophilia A mice after platelet-specific FVIII gene therapy is CD4 T cell-mediated and the immune tolerance is transferable (34). Furthermore, HSCs that are genetically modified to promote platelet-specific FVIII expression can successfully engraft even in a setting of pre-existing anti-FVIII immunity, resulting in sustained therapeutic expression of platelet-FVIII that is coupled with loss of anti-FVIII antibodies (35–37). In contrast, when FVIII expression is targeted to endothelial cells under control of the endothelial cell-specific Tie-2 promoter in a transgenic model, plasma levels of FVIII was fully normalized, but robust immune responses against FVIII occurred, and plasma FVIII dropped to undetectable levels when animals were immunized with FVIII (38).

The tolerance that results with neo-FVIII expressed in platelets of FVIII-deficient mice led us to question whether this process is specific for a coagulant factor or whether it represents a general tolerance mechanism associated with our platelet-targeted gene therapy approach. Further it was of interest to determine the specific tolerogenic mechanisms that drive tolerance induction when platelets are engineered to deliver neo-proteins as a form of gene therapy. To this end, we used ovalbumin (OVA) as a surrogate non-coagulant protein and constructed a lentiviral vector in which OVA is driven by the same αIIb promoter that we used for FVIII and FIX studies (31–37, 39). We show that protein delivery by platelet-based gene therapy effectively utilizes multiple peripheral tolerance mechanisms and promotes profound immune tolerance to neo-proteins.

## Results

### Targeted expression of OVA-encoding gene with the platelet-specific αIIb promoter into HSCs results in its storage in the α-granules of platelets

**Figure 1:**
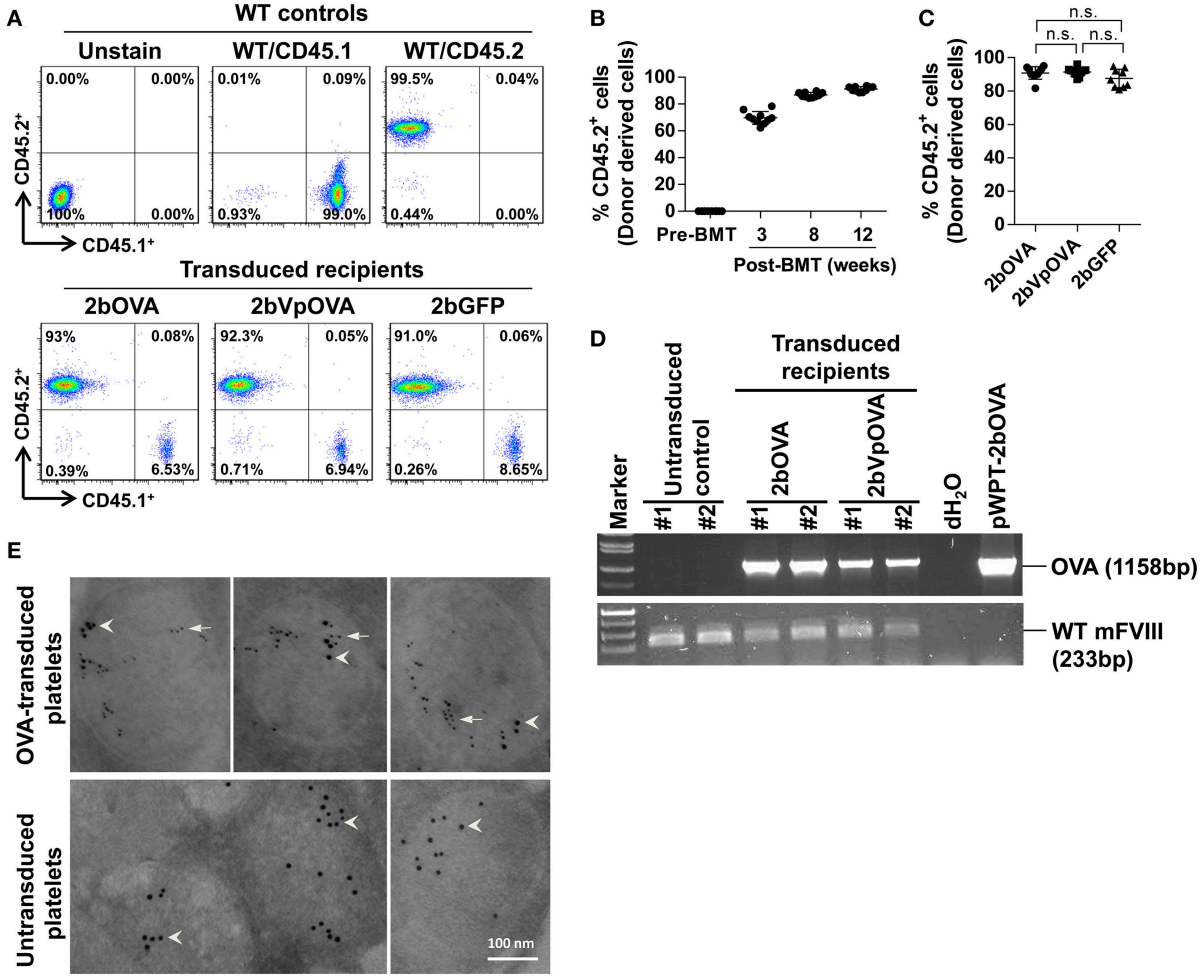
Platelet-targeted OVA gene transfer results in OVA expressed and stored in platelet α-granules. Sca-1^+^ cells isolated from wild type (WT)/CD45.2^+^ donors were transduced with 2bOVA or 2bVpOVA lentivirus and transplanted into WT/CD45.1^+^ recipients that were preconditioned with 660 cGy total body irradiation. The 2bGFP lentiviral vector was used as a control in parallel. Blood samples were collected from recipients starting at 3 weeks after transplantation for analysis. Chimerism was analyzed by flow cytometry. Intracellular location of OVA protein was determined by immunoelectron electron microscopy (EM). Samples from WT/CD45.1 and WT/CD45.2 untreated animals were used as controls for assays. **(A)** Representative dot plots from flow cytometry analysis of chimerism 12 weeks post transplantation. Cells were stained for CD45.1, CD45.2, CD4, and CD8. After staining, cells were analyzed by flow cytometry. DAPI was used to exclude dead cells. **(B)** Chimerism in the 2bOVA group over time after bone marrow transplantation. **(C)** Chimerism in indicated groups at 16 weeks after transplantation. **(D)** PCR analysis shows that OVA proviral DNA was detected in 2bOVA- and 2bVpOVA-transduced recipients. WT mouse FVIII was used as an internal control. **(E)** Intracellular location of OVA determined by EM study. Transgene protein OVA was labeled with small colloidal gold as some indicated by arrows. Endogenous protein VWF was labeled with large colloidal gold as some indicated by arrow heads.

To target OVA expression in platelets, we constructed a lentiviral vector (LV) in which OVA is driven by the αIIb promoter (2bOVA) (Figure 1 Supplemental Figure 1A). Since von Willebrand factor (VWF) propeptide (Vp) can reroute unrelated secretory proteins to a storage pathway,(40) we designed a second vector, 2bVpOVA (Figure 1 Supplemental Figure 1B), which contains Vp to secure OVA storage in platelet granules. We examined the capacity of 2bOVA or 2bVpOVA lentiviral gene delivery to HSCs to express OVA in WT C57BL6 mice. Sca-1^+^ cells from wild type (WT)/CD45.2 mice were transduced with 2bOVA or 2bVpOVA LV and transplanted into WT/CD45.1 recipients preconditioned with 660 cGy total body irradiation (TBI). 2bGFP (Figure 1 Supplemental Figure 1C), in which GFP expression was directed by the same αIIb promoter, was used as a control vector. Following transplantation of transduced cells, engraftment gradually increased from around 60% at week 3 to 90% at week 12 (Figures 1A,B). Bone marrow (BM) of the recipient mice were fully reconstituted by 12 weeks (Figure 1B), with similar levels of engraftment among the three groups (89 ± 2%, 86 ± 5%, and 84 ± 7% in the 2bOVA, 2bVpOVA, and 2bGFP groups, respectively) (Figure 1C).

To confirm viable engraftment of OVA-transduced HSCs, we used PCR to detect OVA proviral DNA in peripheral leukocytes. The OVA cassette was detected in all of the animals that received 2bOVA- or 2bVpOVA-transduced cells (Figure 1D). To determine the intracellular location of the OVA transgene protein, we employed immunogold labeling and electron microscopy. OVA protein was detected in OVA-transduced platelets using either the 2bOVA or the 2bVpOVA vector and was located within the α-granules of platelets. As an internal control, VWF, an endogenous protein that localizes within platelet α-granules, was detected in both transduced and untransduced platelets (Figure 1E).

**Figure 2:**
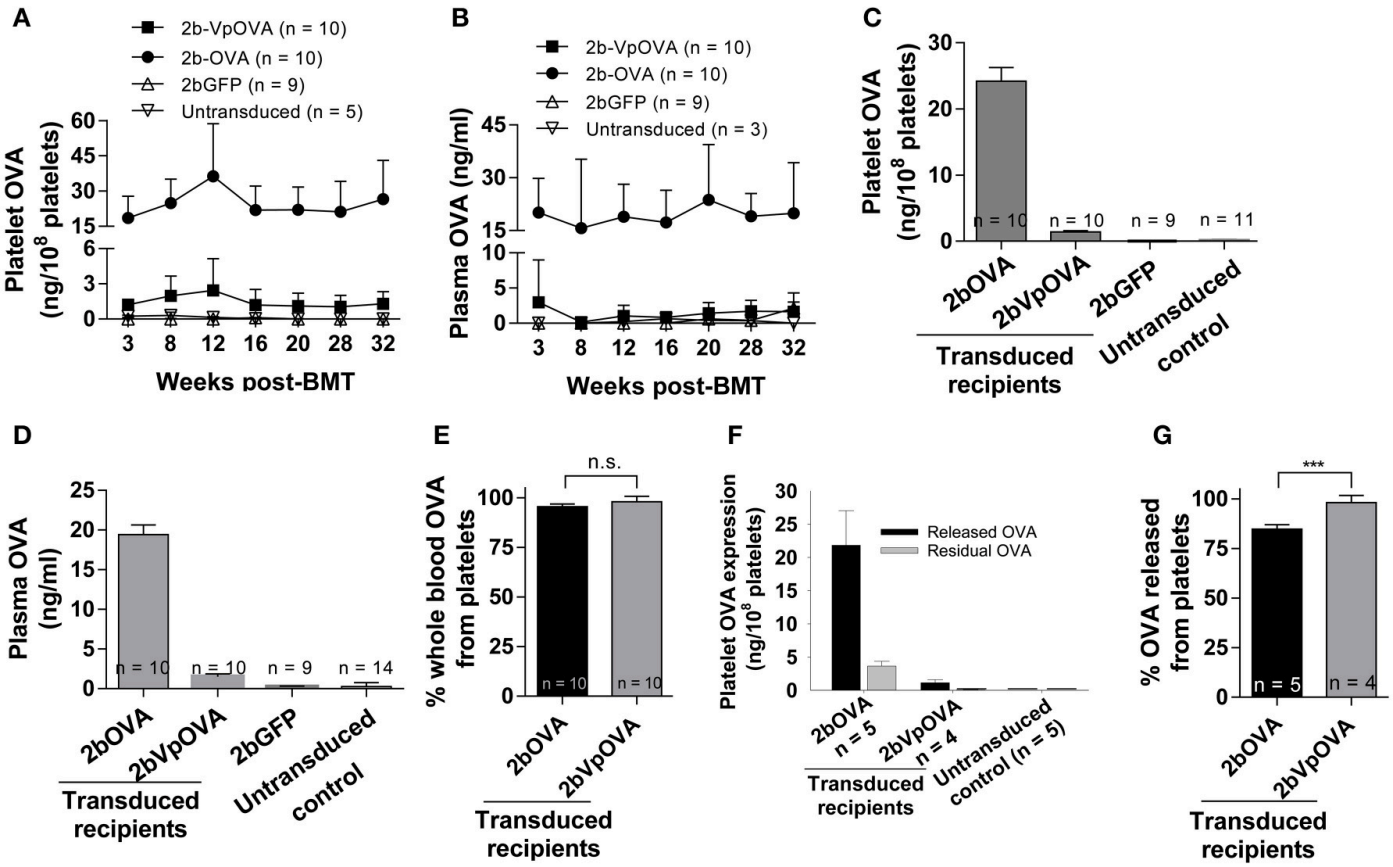
OVA expression levels in transduced recipients. Blood samples were collected from recipients monthly after transplantation and OVA expression was quantified by ELISA assay on platelet lysates, platelet releasates, and plasmas. **(A)** Average expression levels of platelet-OVA at each time point. **(B)** Average expression levels of plasma-OVA at each time point. **(C)** Average expression levels of platelet-OVA over the study period. For individual mice analyzed more than once over the study, the average platelet OVA was calculated. **(D)** Average expression levels of plasma-OVA over the study period. For individual mice analyzed more than once over the study, the average plasma OVA was calculated. **(E)** OVA distribution in whole blood in recipients. Greater than 95% OVA was stored in platelets. The unpaired student *t*-test was used to compare the two groups. “n.s.” indicates no statistically significant difference between the two groups. **(F)** OVA expression levels in platelet releasates. **(G)** Percentage of OVA released from platelets upon agonist stimulation. The unpaired student *t*-test was used to compare the two groups. ^***^P < 0.001. Data shown were summarized from two independent experiments. Data were expressed as the mean ± SD.

Next, we determined the levels of OVA protein in both platelets and plasma at various time points after transplantation by ELISA. Sustained OVA expression was observed in transduced recipients in both 2bOVA and 2bVpOVA-transduced groups with a 17-fold higher platelet-OVA expression in the 2bOVA group compared to the 2bVpOVA group (Figures 2A,B). No OVA was detected in 2bGFP-transduced recipients or untransduced control animals, as expected. When the distribution of OVA in whole blood were calculated, approximately 95% and 98% of OVA protein in whole blood was stored in platelets with an average OVA protein level of 24.22 ± 8.72 ng/10^8^ and 1.41 ± 0.73 ng/10^8^ platelets in 2bOVA and 2bVpOVA transduced recipients, respectively (Figures 2C–E). To investigate whether stored OVA in transduced platelets is released following platelet activation, isolated platelets were stimulated with an agonist cocktail, and OVA levels in platelet releasates and lysates from residual pellets were quantified by ELISA. As shown in Figures 2F–G, 84.8 ± 2.3% and 98.2 ± 3.6% of OVA in platelets were released in the 2bOVA and 2bVpOVA groups, respectively.

Taken together, these data demonstrate that platelet-targeted OVA gene transfer by lentiviral gene delivery to HSCs can efficiently introduce OVA expression and storage in platelet α-granules in peripheral blood and that OVA is released upon platelet activation *in vitro*. The continued presence of plasma OVA also indicated that immune clearance was minimal. This was investigated further.

### Platelet-specific OVA gene delivery into HSCs induces immune tolerance

**Figure 3:**
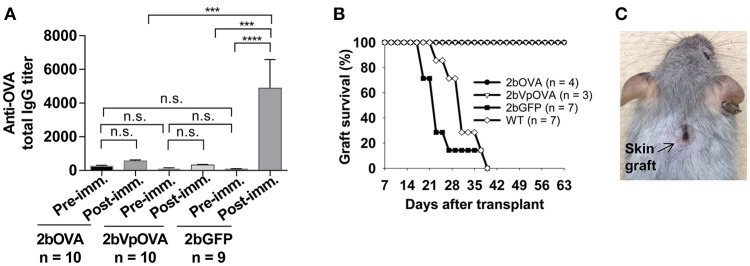
Lentivirus-mediated platelet-specific OVA gene delivery to HSCs induces immune tolerance to OVA. To study if immune tolerance is induced after platelet-targeted OVA gene transfer, anti-OVA antibodies in CD45.1^+^/WT recipients that received 2bOVA- or 2bVpOVA-transduced CD45^+^/WT transduced Sca-1^+^ cells were monitored after gene transfer. Animals that received 2bGFP-transduced Sca-1^+^ cells were used as a control in parallel. Five months post-transplantation, animals were challenged with OVA to investigate whether immune tolerance was induced in platelet-targeted OVA-transduced recipients. The titers of anti-OVA total IgG were determined by ELISA assay. Some animals were transplanted with the skin graft from CAG-OVA transgenic mice, in which OVA is expressed on the surface of all cells. Animals were monitored for skin graft acceptance, and complete graft rejection was recorded. **(A)** Anti-OVA total IgG titers. Data are presented as mean ± SD and analyzed by one way ANOVA. “n.s.” indicates no statistically significant difference between the two groups. ^***^P < 0.001 and ^****^P < 0.0001. **(B)** Skin graft survival rate. **(C)** Representative skin graft on the 2bOVA-transduced recipient (6 months after skin transplantation).

To investigate whether anti-OVA immune tolerance was induced in recipients after platelet-specific OVA gene transfer, anti-OVA antibody titers were monitored in transduced recipients before and after OVA immunization (Figure 3A). There were no statistically significant differences in the titers of OVA-specific IgG at 16 weeks after transplantation among the three groups before or the 2bOVA and the 2bVpOVA groups after OVA immunization. In contrast, the titer of OVA-specific IgG antibodies in the 2bGFP control group significantly increased from 84 ± 50 to 4,889 ± 5,079 after OVA challenge. Although the total OVA expression level in the 2bOVA group was 17-fold higher than in the 2bVpOVA group, the titer of anti-OVA total IgG in the 2bVpOVA group was not significantly different than that in the 2bOVA group. These data demonstrate that HSC-based gene delivery that targets the synthesis of the surrogate gene therapy product OVA specifically within platelets under control of the αIIb promoter can suppress the anti-OVA antibody response following primary immunization.

To explore whether platelet targeted gene transfer can be applied to prevent a T cell-mediated immune response, skin transplantation was performed. Skin grafts from CAG-OVA transgenic mice, in which OVA is expressed under control of the chicken beta-actin promoter and detected in all tissues (41), were transplanted onto transduced recipients. Full-thickness tail skin successfully grafted onto 2bOVA- or 2bVpOVA-transduced recipients and was sustained during the study course (6 months post-transplantation). In contrast, skin grafts were completely rejected in untransduced WT and 2bGFP-transduced animals within 6 weeks (Figures 3B,C).

Collectively, these results demonstrate that targeting transgene expression to platelets can efficiently induce immune tolerance to the targeted protein.

### Clonal deletion of antigen-specific CD4 T cells occurs in peripheral lymphoid organs after platelet-specific OVA gene delivery into HSCs

**Figure 4:**
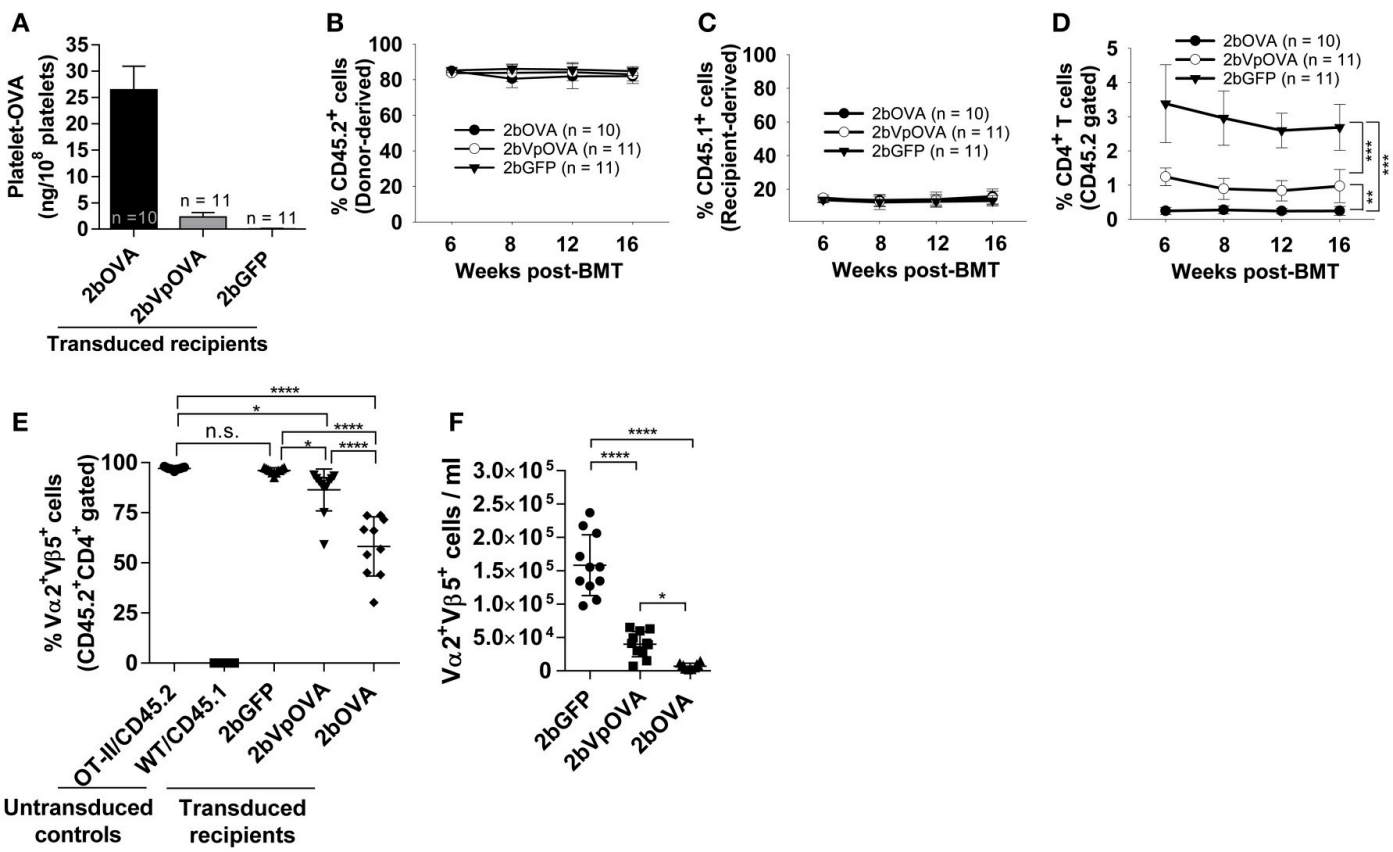
Targeting OVA expression to platelets results in OVA-specific CD4 T cell deletion in peripheral blood. To study the mechanisms by which immune tolerance is established after platelet-targeted OVA gene transfer, Sca-1^+^ cells from OVA-specific TCR transgenic mice (OT-II/CD45.2) were transduced with 2bOVA or 2bVpOVA lentivirus and transplanted into WT/CD45.1 recipients that were preconditioned with 660 cGy total body irradiation. Animals were analyzed by ELISA for OVA expression and flow cytometry for chimerism and OVA-specific CD4 T cells in peripheral blood. For chimerism analysis, cells were stained with CD45.1, CD45.2, CD4, and CD8 antibodies. For Vα2Vβ5 analysis, cells were stained with CD45.2, CD4, Vα2TCR, and Vβ5TCR antibodies. After staining, cells were analyzed by flow cytometry. DAPI was used to exclude dead cells. Samples from OT-II/CD45.2 and WT/CD45.1 untreated animals were used as controls. **(A)** Average expression levels of platelet-OVA over the study period. For individual mice analyzed more than once over the study, the average platelet OVA was calculated. **(B)** Average donor (OT-II/CD45.2)-derived cell percentage (chimerism) at each time point. **(C)** Average percentage of recipient (WT/CD45.1)-derived cells. **(D)** Average percentage of OVA-specific CD4 T cells among donor-derived leukocytes at each time point. **(E)** Percentage of OVA-specific Vα2^+^Vβ5^+^ cells in donor-derived CD4 T cells in peripheral blood (3 months post-BMT). **(F)** Total number of OVA-specific Vα2^+^Vβ5^+^ cells in peripheral blood (3 months post-BMT). Data shown were summarized from two independent experiments. Data were expressed as the mean ± SD. Statistical comparisons of experimental groups were evaluated by the one way ANOVA. ^*^P < 0.05; ^**^P < 0.01; and ^***^P < 0.001. “n.s.” indicates no statistically significant difference between the two groups.

To explore how immune tolerance is established following platelet-specific gene expression, we transduced Sca-1^+^ cells from OT-II/CD45.2 transgenic mice (42), in which 98% of CD4^+^ T cells express major histocompatibility complex (MHC) class II-restricted OVA_323−339_-specific Vα2Vβ5 TCR (T cell receptor), with lentivirus encoding 2bOVA, 2bVpOVA, or 2bGFP and transplanted into WT/CD45.1 recipients preconditioned with 660 cGy TBI. After transplantation and BM reconstitution, platelet-OVA expression were observed in the recipients that received either 2bOVA- or 2bVpOVA-transduced OT-II/CD45.2 cells (26.48 ± 4.47 ng/10^8^ platelets and 2.31 ± 0.81 ng/10^8^ platelets, respectively, Figure 4A). This is similar to the levels observed in 2bOVA- or 2bVpOVA-transduced WT/CD45.2 cells (Figure 2C). Thus, the expression of OVA-specific T cells did not affect platelet production of neo-protein OVA, possibly indicative of tolerance.

**Figure 5:**
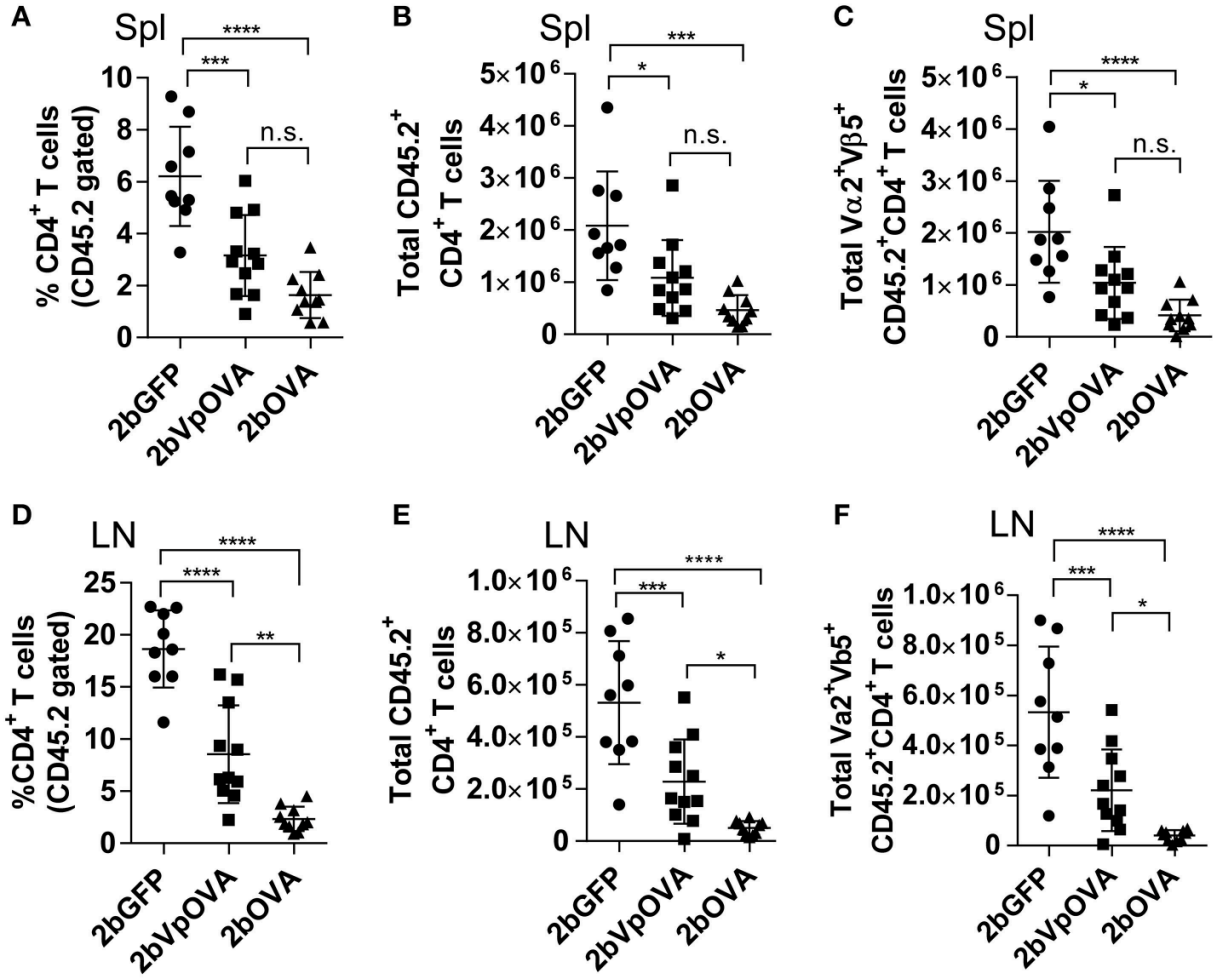
Targeting OVA expression to platelets results in OVA-specific CD4 T cell deletion in periphery lymphoid organs. Five months after transplantation of OVA-transduced Sca-1^+^ OT-II/CD45.2 cells, animals were sacrificed. Spleens (Spl) and lymph nodes (LN) were isolated from transduced recipients and single cell suspensions were prepared. One million cells were stained with CD45.1, CD45.2, CD4, Vα2TCR, and Vβ5TCR antibodies. After staining, cells were analyzed by flow cytometry. DAPI was used to exclude dead cells. Samples from OT-II/CD45.2 and WT/CD45.1 untreated mice were used as controls. **(A)** Percentage of CD4 T cells among donor (OT-II/CD45.2)-derived leukocytes in spleen. **(B)** Total donor-derived CD4 T cells in spleen. **(C)** Total donor-derived OVA-specific (Vα2^+^Vβ5^+^) CD4 T cells in spleen. **(D)** Percentage of CD4 T cells among donor (OT-II/CD45.2)-derived leukocytes in lymph nodes. **(E)** Total donor-derived CD4 T cells in lymph nodes. **(F)** Total donor-derived OVA-specific CD4 T cells in lymph nodes. Data shown were summarized from two independent experiments. Data were expressed as the mean ± SD. Statistical comparisons of experimental groups were evaluated by the one way ANOVA. ^*^P < 0.05; ^**^P < 0.01; ^***^P < 0.001; and ^****^P < 0.0001. “n.s.” indicates no statistically significant difference between the two groups.

The engraftments among the three groups were comparable both in term of donor-derived (Figure 4B) and remaining recipient cells (Figure 4C and Figure 4 Supplemental Figure 1). However, average donor-derived CD45.2^+^CD4^+^ T cells during the study course in the 2bOVA (0.25 ± 0.10%) and 2bVpOVA groups (0.94 ± 0.37%) were significantly lower than in the 2bGFP group (2.90 ± 0.79%) in peripheral blood during the study course (Figure 4D). The frequency and the total number of OVA-specific Vα2^+^Vβ5^+^ CD4^+^ T cells in both the 2bOVA and 2bVpOVA groups were significantly lower than in the 2bGFP group (Figures 4E,F), indicating that CD4^+^ T cells with Vα2^+^Vβ5^+^ expression are susceptible to deletion after platelet-specific OVA expression in a dose dependent manner. When CD4^+^ T cells in lymphoid organs were analyzed at 5 months after transplantation, similar to the data obtained from peripheral blood, the frequency and the total number of donor-derived CD45.2^+^CD4^+^ T cells in both spleen and lymph nodes were significantly lower in the 2bOVA and 2bVpOVA groups compared to the 2bGFP group (Figures 5A–F). The frequency and the total number of donor-derived CD45.2^+^CD4^+^ T cells in lymph nodes in the 2bOVA group were significantly lower than the 2bVpOVA group (Figures 5D–F), but there were no differences in spleen between the two groups (Figures 5A–C).

**Figure 6:**
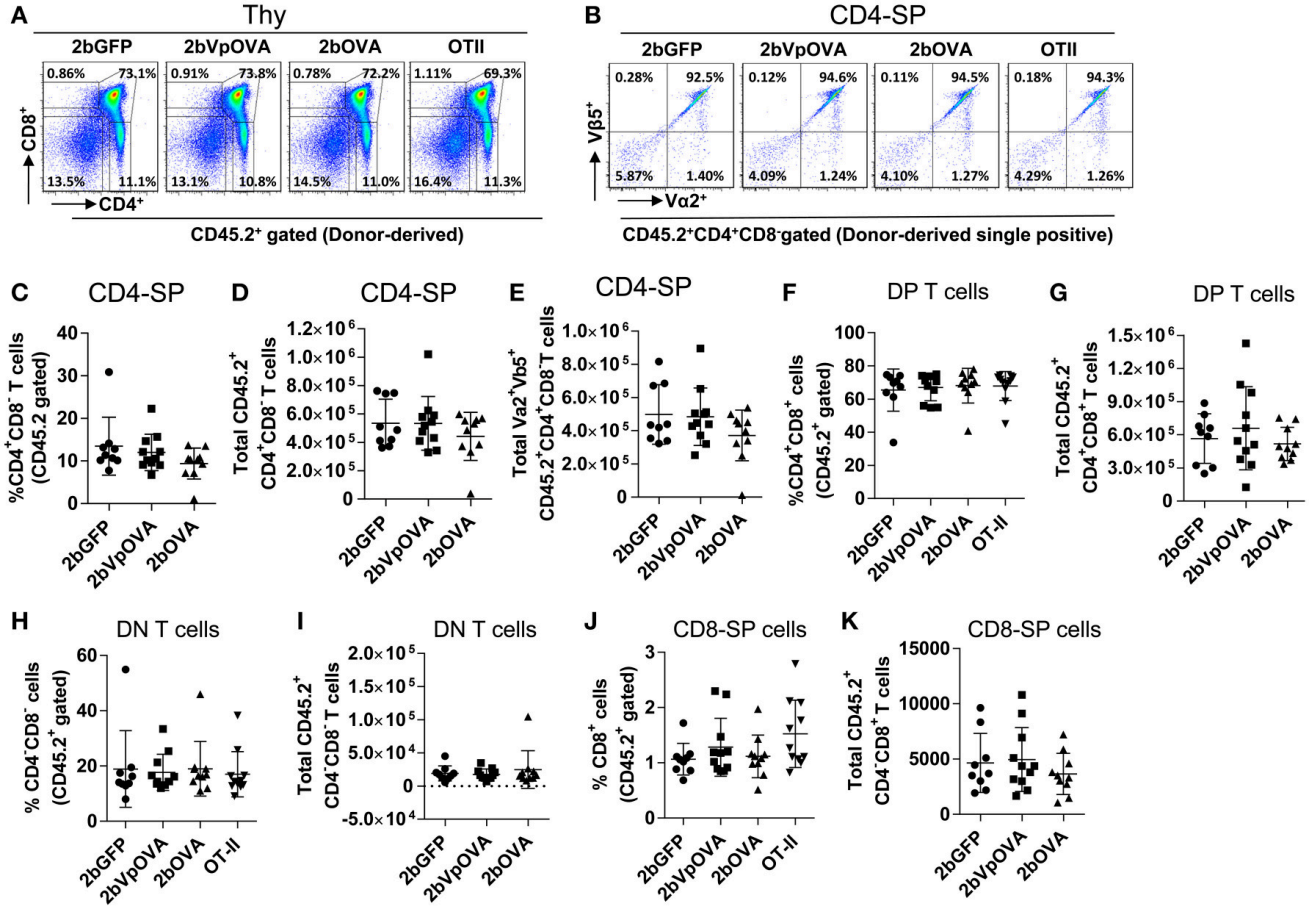
OVA-specific thymocytes are untouched after targeting OVA expression to platelets. Five months after BMT, animals were sacrificed. Cells were isolated from thymus (Thy) and stained with CD45.1, CD45.2, CD4, CD8, Vα2TCR, and Vβ5TCR antibodies. After staining, cells were analyzed by flow cytometry. DAPI was used to exclude dead cells. Samples from OT-II/CD45.2 and WT/CD45.1 untreated mice were used as controls. Donor-derived (CD45.2^+^) leukocytes were gated and analyzed for CD4 and CD8 T cells. **(A)** Representative dot plots of donor-derived T cells in thymus. **(B)** Representative dot plots of Vα2Vβ5 staining of donor-derived single positive (CD4-SP) T cells in thymus. **(C)** Percentage of donor-derived single positive CD4 T cells in thymus. **(D)** Total donor-derived single positive CD4 (CD4-SP) T cells in thymus. **(E)** Total donor-derived OVA-specific CD4-SP T cells in thymus **(F)** Percentage of double positive (DP) (CD4^+^CD8^+^) T cells among donor-derived (CD45.2^+^) cells in thymus. **(G)** Total number of donor-derived double positive T cells in thymus. **(H)** Percentage of double negative (DN) (CD4^−^CD8^−^) T cells among donor-derived (CD45.2^+^) cells in thymus. **(I)** Total number of donor-derived double negative T cells in thymus. **(J)** Percentage of CD8 single positive (CD8-SP) T cells among donor-derived leukocytes in thymus. **(K)** Total number of donor-derived CD8-SP T cells in thymus. Data shown were summarized from two independent experiments. Data were expressed as the mean ± SD. Statistical comparisons of experimental groups were evaluated by the one way ANOVA and there are no statistically significant differences among groups.

**Figure 7:**
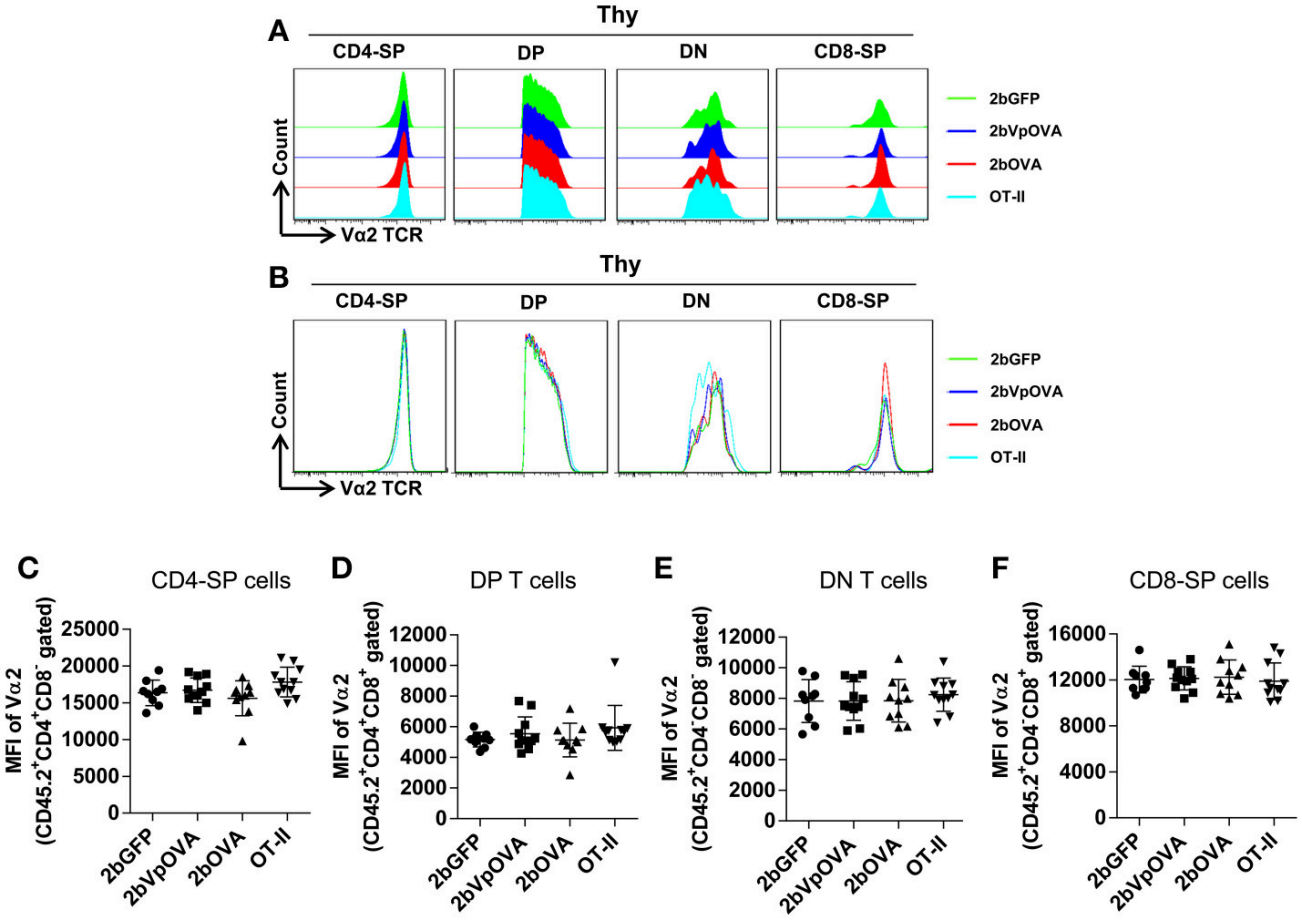
Flow cytometry analysis of the clonotype TCR Vα2 in donor-derived thymocytes. Five months after BMT, animals were sacrificed. Cells were isolated from thymus (Thy) and stained with CD45.1, CD45.2, CD4, CD8, Vα2TCR, and Vβ5TCR antibodies. After staining, cells were analyzed by flow cytometry. Donor-derived (CD45.2^+^) leukocytes were gated and analyzed for CD4 and CD8 T cells. Various compartments: CD4 single positive (CD4-SP); CD4 and CD8 double positive (DP) T cells; CD4 and CD8 double negative (DN) T cells; and CD8 single positive (CD8-SP) were gated separately and analyzed for TCR Vα2 expression. **(A)** Representative histograms from various groups. **(B)** Merged histograms from various groups. **(C)** The MFI of TCR Vα2 expression in donor-derived CD4-SP compartment in thymus. **(D)** The MFI of TCR Vα2 expression in donor-derived double positive T cell compartment in thymus. **(E)** The MFI of TCR Vα2 expression in donor-derived double negative T cell compartment in thymus. **(F)** The MFI of TCR Vα2 expression in donor-derived CD8-SP compartment in thymus. Data shown were summarized from two independent experiments. Data were expressed as the mean ± SD. Statistical comparisons of experimental groups were evaluated by the one way ANOVA and there are no statistically significant differences among groups.

The reduced number of antigen-specific CD4 cells in the periphery could be the result of central tolerance deleting the number mature thymocytes able to populate the periphery. This would be compatible with central tolerance mechanism. Interestingly, there were no differences in either the percentage or the total cell number of donor-derived single positive CD4^+^ T cells (CD4-SP, CD45.2^+^CD4^+^CD8^−^) (Figures 6A–E), double positive (DP, CD45.2^+^CD4^+^CD8^+^) (Figures 6F–G), double negative (DN, CD45.2^+^CD4^−^CD8^−^) (Figures 6H–I), or single positive CD8^+^ T cells (CD8-SP, CD45.2^+^CD4^−^CD8^+^) (Figures 6J,K) in the thymus among the 2bOVA, 2bVpOVA, and 2bGFP groups. The percentage and total number of OVA-specific Vα2^+^Vβ5^+^ CD4^+^CD8^−^ T cells in the thymus of 2bVpOVA-transduced and 2bOVA-transduced recipients were comparable to those in 2bGFP-transduced recipients (Figures 6B,E). To investigate whether the T cell clonotype changes after platelet-specific OVA gene transfer, we further analyzed the Vα2^+^ TCR train expression. The expression of donor-derived Vα2^+^ cells in the thymus was similar among the 3 groups as shown in representative flow cytometry histograms (Figures 7A,B) and the MFI (mean fluorescence intensity) (Figures 7C–F), indicating that there was not apparent clonotype rearrangement in thymus after platelet-targeted OVA gene transfer. These data demonstrate that central tolerance may not play a role in platelet-targeted gene therapy. Of note, the frequency and total number of the remaining endogenous CD4^+^ T cells were similar among the 3 groups in peripheral blood and all lymphoid organs (Figure 5 Supplemental Figures 1A–G).

**Figure 8:**
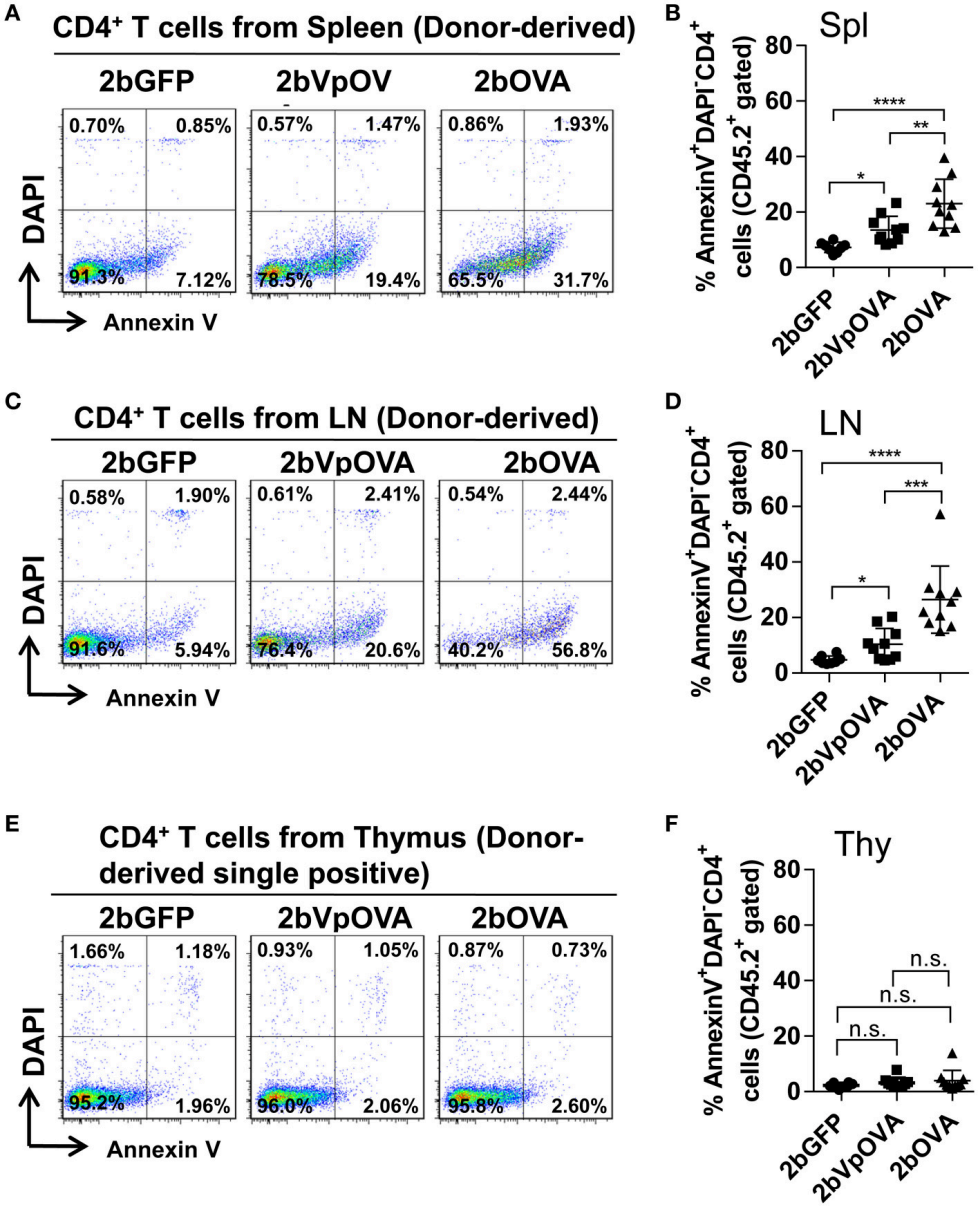
Platelet-targeted OVA expression results in OVA-specific CD4^+^ T cells undergoing apoptosis in spleen and lymph nodes, but not in thymus. Five months after transplantation of OVA-transduced Sca-1^+^ OT-II/CD45.2 cells, animals were sacrificed. One million cells from spleens, lymph nodes, and thymi were stained with CD45.1, CD45.2, CD4, Annexin V, and DAPI. After staining, cells were analyzed by flow cytometry. Cells from OT-II/CD45.2 and WT/CD45.1 mice were used as controls in parallel for staining. **(A)** Representative dot plots from flow cytometry analysis of donor (OT-II/CD45.2)-derived apoptotic CD4^+^ T cells in spleen. **(B)** Percentage of donor-derived apoptotic CD4^+^ T cells in spleen. **(C)** Representative dot plots from flow cytometry analysis of donor-derived apoptotic CD4^+^ T cells in lymph nodes. **(D)** Percentage of donor-derived apoptotic CD4^+^ T cells in lymph nodes. **(E)** Representative dot plots from flow cytometry analysis of donor-derived apoptotic single positive CD4 T cells in thymus. **(F)** Percentage of donor-derived apoptotic single positive CD4 T cells in thymus. Data shown were summarized from two independent experiments. Data were expressed as the mean ± SD. Statistical comparisons of experimental groups were evaluated by the one way ANOVA. ^*^P < 0.05; ^**^P < 0.01; ^***^P < 0.001; and ^****^P < 0.0001. “n.s.” indicates no statistically significant difference between the two groups.

Since the lack of peripheral antigen-specific CD4 T cells does not appear to be due to central tolerance, we investigated whether there was evidence for apoptotic cell loss in the periphery. To this end, we stained cells with Annexin V, which revealed that the percentage of apoptotic CD45.2^+^CD4^+^ T cells in the 2bOVA and 2bVpOVA groups were significantly higher than in the 2bGFP group in both spleen (23.0 ± 8.80%, 13.51 ± 4.91%, and 7.29 ± 1.84%, respectively) (Figures 8A,B) and lymph nodes (26.45 ± 12.11%, 10.32 ± 5.69%, and 4.72 ± 1.48%, respectively) (Figures 8C,D), but not in the thymus (3.97 ± 3.64%, 3.17 ± 1.79%, and 2.22 ± 0.88%, respectively) (Figures 8E,F). There were no statistically significant differences in the percentage of apoptotic CD45.1^+^CD4^+^ T cells (recipient-derived) among the three groups in spleen, lymphoid nodes, and thymus (Figure 8 Supplemental Figure 1).

Together, these data demonstrate that antigen-specific CD4^+^ T cells are deleted in peripheral lymphoid organs after platelet-specific gene transfer, compatible with a peripheral tolerance mechanism.

### Antigen-specific regulatory T (Treg) cells are increased in peripheral lymphoid organs after platelet-targeted OVA gene transfer

**Figure 9:**
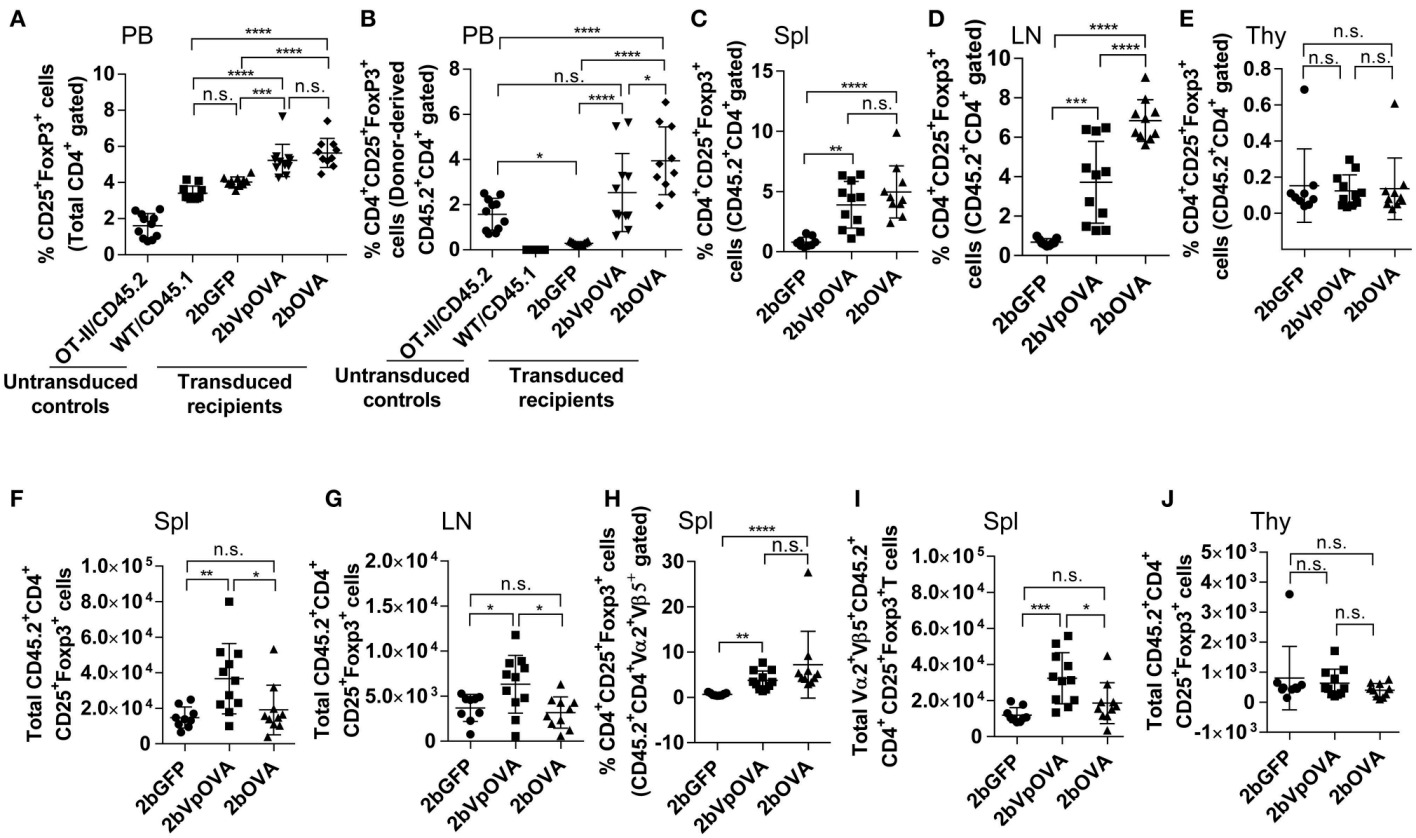
Platelet-targeted OVA expression results in Treg cell induction. Leukocytes from peripheral blood (4 months post-BMT of OVA-transduced Sca-1^+^ OT-II/CD45.2 cells) or lymphoid organs (5 months post-BMT) were stained with CD45.1, CD45.2, CD4, CD25, and Foxp3 antibodies. Samples from OT-II/CD45.2 mice and WT/CD45.1 mice were used as controls for staining in parallel. After staining, cells were analyzed by flow cytometry. **(A)** Percentage of Treg cells (CD25^+^Fopx3^+^) among total CD4 T cells in peripheral blood. **(B)** Percentage of Treg cells among donor (OT-II/CD45.2)-derived CD4 T cells in peripheral blood. **(C)** Percentage of Treg cells among donor-derived CD4 T cells in spleen. **(D)** Percentage of Treg cells among donor-derived CD4 T cells in lymph nodes. **(E)** Percentage of Treg cells among donor-derived CD4 T cells in thymus. **(F)** Total number of donor-derived Treg cells in spleen. **(G)** Total number of donor-derived Treg cells in lymph nodes. **(H)** Percentage of Treg cells among donor-derived OVA-specific CD4 (CD45.2^+^CD4^+^Vα2^+^Vβ5^+^) T cells in spleen. **(I)** Total number of donor-derived OVA-specific Treg (CD45.2^+^CD4^+^CD25^+^Foxp3^+^Vα2^+^Vβ5^+^) cells in spleen. **(J)** Total number of donor-derived Treg cells in thymus. Data shown were summarized from two independent experiments. Data were expressed as the mean ± SD. Statistical comparisons of experimental groups were evaluated by the one way ANOVA. ^*^P < 0.05; ^**^P < 0.01; ^***^P < 0.001; and ^****^P < 0.0001. “n.s.” indicates no statistically significant difference between the two groups.

Since Treg cells are also modulators of immune tolerance, we evaluated their abundance in OVA-transduced recipients. The frequencies of total regulatory T (Treg) cells in peripheral blood in the 2bOVA and 2bVpOVA groups (5.64 ± 0.81% and 5.23 ± 0.89%, respectively) were significantly higher than in the 2bGFP group (4.04 ± 0.27%) (Figure 9A and Figure 9 Supplemental Figure 1). Likewise, the frequencies of donor-derived Treg cells in the 2bOVA and 2bVpOVA groups were significantly higher than in the 2bGFP group in peripheral blood (3.95 ± 1.50%, 2.54 ± 1.73%, and 0.29 ± 0.08%, respectively) (Figure 9B), spleen (4.97 ± 2.17%, 3.90 ± 1.94%, and 0.80 ± 0.40%, respectively) (Figure 9C), and lymph nodes (6.85 ± 1.06%, 3.72 ± 2.07%, and 0.67 ± 0.17%, respectively) (Figure 9D), but not in the thymus (0.14 ± 0.17%, 0.12 ± 0.09%, and 0.15 ± 0.20%, respectively) (Figure 9E).

The total number of donor-derived Treg cells in the 2bVpOVA group was significantly higher than in the 2bGFP and 2bOVA groups in spleen and lymph nodes, but there was no statistical difference between the 2bOVA and 2bGFP groups (Figures 9F–G). When donor-derived OVA-specific Treg cells (CD45.2^+^CD4^+^CD25^+^FoxP3^+^Vα2^+^Vβ5^+^) in spleen were further analyzed, both the frequency and the total number (Figures 9H–I) in the three groups were similar to those obtained using the markers of donor-derived Treg (CD45.2^+^CD4^+^CD25^+^FoxP3^+^) cells (Figures 9C–F), confirming that donor-derived Treg cells were OVA-specific. There were no differences in the total number of Treg cells among the three groups in the thymus (Figure 9J). There were no statistically significant differences in recipient-derived Treg (CD45.1^+^CD4^+^CD25^+^Foxp3^+^) cells regardless of frequency or total number among the three groups in peripheral blood (Figure 9 Supplemental Figure 2A), spleen (Figure 9 Supplemental Figures 2B,C), lymph nodes (Figure 9 Supplemental Figures 2D–E), and thymus (Figure 9 Supplemental Figures 2F–G).

**Figure 10:**
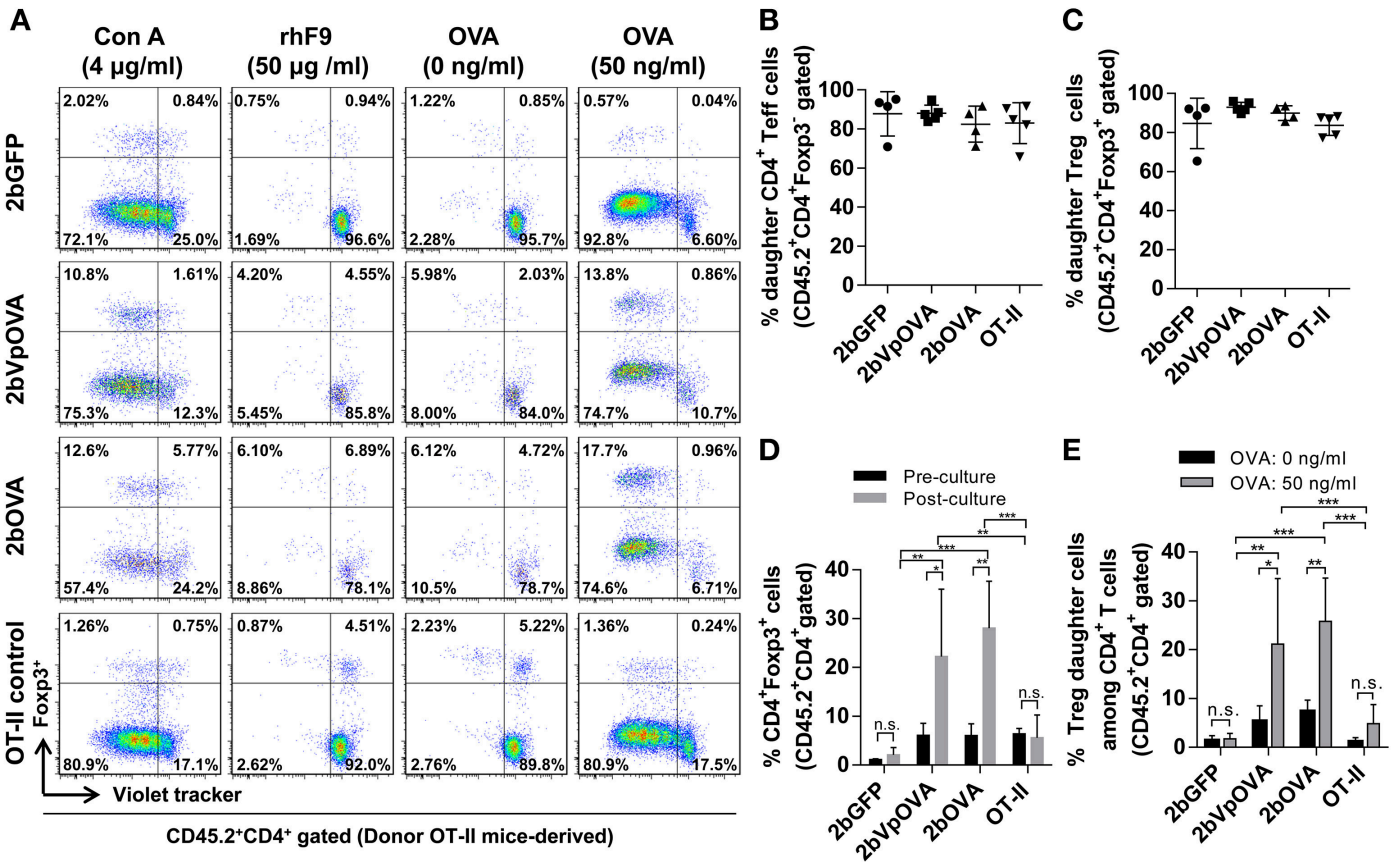
Treg cells induced in recipients after platelet-targeted OVA expression suppressed effector T cell (Teff) proliferation in response to OVA stimulation *in vitro*. Five months after transplantation of OVA-transduced Sca-1^+^ OT-II/CD45.2 cells, animals were sacrificed. Spleen was isolated, grinded, and single cell suspension was prepared. Splenocytes were labeled with CellTrace Violet and cultured with or without OVA for 96 h. Cells were stained for mouse CD45.2, CD4, Foxp3 and analyzed by flow cytometry. Recombinant human factor IX (rhF9) was used as an unrelated antigen control. Con A was used as a positive control for CD4 T cell proliferation. **(A)** Representative dot plots of Treg and non-Treg (CD4^+^ T effector [Teff]) cell proliferation in response to OVA stimulation *in vitro*. **(B)** Percentage of donor-derived daughter CD4 Teff cells. **(C)** Percentage of donor-derived daughter Treg cells. **(D)** The percentage of Treg cells among donor-derived CD4 T cells before and after OVA stimulation *in vitro*. **(E)** Percentage of donor-derived daughter Treg cells among donor-derived CD4 T cells after OVA stimulation *in vitro*. Data were expressed as the mean ± SD. Statistical comparisons of experimental groups were evaluated by the one way ANOVA. ^*^P < 0.05; ^**^P < 0.01; and ^***^P < 0.001. “n.s.” indicates no statistically significant difference between the two groups.

To investigate how donor-derived Treg and non-Treg CD4^+^ T (effector T [Teff]) cells in OVA-transduced recipients respond to OVA stimulation, splenocytes were isolated and cultured for 96 h with or without OVA. When splenocytes were stimulated with OVA *in vitro*, both donor-derived Treg cells (CD45.2^+^CD4^+^Foxp3^+^) and CD4^+^ Teff (CD45.2^+^CD4^+^Foxp3^−^) cells proliferated significantly in contrast to those without OVA stimulation, or to those with the unrelated antigen recombinant FIX (rhF9) stimulation (Figure 10A). There were no statistically significant differences in the percentage of daughter CD4^+^ Teff or Treg cells among the 2bOVA, 2bVpOVA, 2bGFP, and OT-II groups (Figures 10B,C). However, after *in vitro* OVA stimulation, the percentage of donor-derived Treg cells in the 2bOVA group and the 2bVpOVA group was significantly increased (3.7- and 4.7-fold) compared to those before culture and was significantly higher than those in the 2bGFP (9.7- and 12.1-fold higher) and OT-II control groups (Figures 10D), demonstrating that proliferated Treg cells in the 2bOVA and 2bVpOVA groups suppressed CD4^+^ Teff cell expansion when OVA is encountered. Furthermore, after OVA stimulation, the percentage of daughter Treg cells among donor-derived CD4^+^ T cells in the 2bOVA group and the 2bVpOVA group significantly increased compared to those without OVA restimulation, or those in the 2bGFP and OT-II control groups (Figures 10E). This proportional increase in Treg cells after OVA stimulation in the co-culture conditions is consistent with OVA-specific Treg cell suppression of CD4^+^ Teff cell proliferation when OVA is encountered, although we did not conduct a direct OVA-specific suppressive functional test for those expanded Treg cells. In contrast, there was no significant difference in the 2bGFP and OT-II groups with or without OVA stimulation. These data demonstrate that OVA-specific Treg cells in 2bOVA- or 2bVpOVA-transduced recipients can specifically respond to OVA stimulation.

Taken together, these results demonstrate that OVA-specific Treg cells are positively selected and/or induced after platelet-specific OVA gene transfer and that OVA-specific Treg cells can expand to suppress CD4^+^ Teff cell proliferation when OVA is encountered.

## Discussion

The central finding of this report is that platelet-specific gene transfer can efficiently induce immune tolerance targeted to proteins expressed in platelet α-granules that are not normally associated with the hemostatic role of platelets. Tolerance involves suppression of specific antibody production and can afford protection from skin graft rejection. Our studies revealed two distinct mechanisms involving peripheral CD4 T cells: clonal deletion of peripheral antigen-specific effector CD4^+^ T cells; and expansion of antigen-specific Treg cells. Despite measurable circulating levels of the neo-protein, there was no evidence for central tolerance mediated by changes in thymic selection. Tolerance to platelet cargo is therefore primarily a robust peripheral process that can be co-opted for use in gene therapy.

Immune responses to neo-proteins can be problematic, reducing the therapeutic efficacy of gene and protein replacement strategies, eliminating biologic therapeutic drugs, and causing graft rejection. Indeed, immune responses against FVIII are a significant concern in both protein replacement therapy and gene therapy of hemophilia A. Taking the advantage of platelets' unique biological capacities as a storage cargo and delivery vehicle (43), we have developed a platelet-specific gene therapy protocol that can efficiently restore hemostasis and engender profound immune tolerance to the therapeutic products in hemophilia models (31–33). Here, we determined that what we observed for FVIII or FIX could be extended to a neo-protein not involved in thrombosis or hemostasis, although it is unclear how neo-proteins traffic into storage granules. This process appears to be independent of adding a chaperone function associated with targeting proteins to the granules. The storage property of platelets is critical for delivery of therapeutic protein for hemostasis in hemophilia models. This feature may also be fundamental in prompting the immune tolerance to neo-protein in platelet-specific gene therapy when gene therapy products are stored together with many cytokines and chemokines, including a significant amount of transforming growth factor-β1 (TGF-β1), a critical immune modulator (43).

In the current study, functional immune tolerance was evidenced by three means. First, transduced recipients were tolerized to 2bOVA- and 2bVpOVA-transduced cells and *de novo* platelet-derived OVA protein after 2bOVA or 2bVpOVA lentiviral gene delivery to HSCs. Secondly, platelet-targeted OVA gene transfer suppressed anti-OVA humoral immune responses even after animals were challenged with exogenous full-length OVA protein. Lastly, the 2bOVA- and 2bVpOVA-transduced recipients were tolerized to the skin graft from CAG-OVA transgenic mice leading to prevention of graft rejection. The specific peripheral tolerogenic mechanisms engaged were related to the level of OVA protein in platelets. At higher levels (the 2bOVA group), clonal deletion of effector T cells was the prominent mechanism. In the 2bVpOVA group, where OVA protein was about 6% of that obtained in the 2bOVA group, clonal deletion was less prominent and expansion of antigen-specific Treg cells was observed. These studies indicate that in our system, the proportional engagement of different peripheral tolerogenic mechanisms is linked to the level of protein expression.

The development of immune tolerance to circulating proteins can involve central and/or peripheral mechanisms (44–46). Our results from the OVA-specific TCR transgenic OT-II mouse model studies demonstrate that the tolerance induced after platelet-specific gene transfer involves the peripheral processes of clonal deletion and Treg cell expansion. Previous studies by Haribhai and colleagues have demonstrated that a low level of a neo-protein circulating in the serum is sufficient to induce a complete central tolerance by clonal deflection of antigen-specific CD4 T cells in thymus. In their model, the neo-protein was expressed in many tissues, including the thymic cortical and medullary epithelium. They showed that partial central tolerance could be achieved even with low serum levels (≤ 0.1 ng/ml) (47). In our platelet-specific OVA model, 95–98% of the OVA protein in blood circulation was stored in platelets, and free OVA in the plasma was approximately 20 ng/ml, which was higher than the threshold reported by Haribhai et al. (47) Interestingly, donor-derived OVA-specific CD4^+^ T cells were largely eliminated by apoptosis in peripheral lymphoid organs, while OVA-specific thymocyte development was intact. Thus transplantation studies, which by design limit any transgene expression to the hematopoietic compartment, indicate that tolerance to platelet cargo is largely a peripheral rather than central process.

Multiple lines of evidence suggest that clearance of apoptotic cells results in immune tolerance induction to the cleared antigens derived from ingested apoptotic cells (48–52). Coupling antigen to apoptotic cells can induce immune tolerance through Treg cell induction (53). In the body, normal physiological aged platelets undergo apoptosis. Thus, the storage of the neo-protein from platelet-specific gene therapy in platelet α-granules together with TGF-β1, which is essential in maintain Treg suppressive function and a critical mediator of iTreg induction, may facilitate induction of antigen-specific Treg cells. Our studies show that OVA-specific Treg cells are maintained and potentially induced. They can expand when needed in platelet-specific OVA-transduced recipients. Expansion of the antigen-specific Treg compartment may complement clonal deletion strategies, which are likely to be incomplete when circulating levels of the target protein are low. Indeed, there were still OVA-specific CD4^+^ Teff cells remaining in 2bVpOVA-transduced recipients. The remaining OVA-specific CD4^+^ Teff cells still reacted to exogenous OVA *in vitro*. Whether the remaining Teff cells after platelet-specific OVA gene transfer are anergic is still unclear and warrants further investigation. Importantly Treg cells from these recipients also proliferated specifically in response to OVA stimulation and suppressed effector CD4^+^ T cell proliferation. These results suggest that there are two distinct mechanisms that doubly secure the immune tolerance in platelet-targeted gene therapy. One is antigen-specific CD4 T cell clone deletion and the other is antigen-specific Treg cell induction/expansion. Our data indicate that overlapping peripheral mechanisms are an essential feature of the robust tolerance that develops in our gene therapy model.

Immune tolerance generated to specific type's self-proteins, such as those contained in platelet granules, is remarkable and of considerable interest when considering the safest approaches to gene therapy. We have revealed two peripheral tolerance mechanisms that are centered on tolerance to proteins expressed in the circulating progeny of megakaryocytes after platelet-specific gene therapy. Data from the current study strongly suggest that platelet-targeted gene therapy can be utilized as an effective approach to induce antigen-specific immune tolerance. It will be interesting to determine if this pathway can be extended to additional non-hemostatic proteins and to targets of auto- or alloimmune responses. In future studies, it will be critical to understand the precise mechanism of the tolerance induction process after platelet-specific gene therapy.

## Materials and methods

### Antibodies and reagents

The following rat anti-mouse monoclonal antibodies (MoAbs) directly conjugated with fluorophore purchased from eBioscience (San Diego, CA) were used in our studies for flow cytometry analysis: CD45.1, CD45.2, CD4, CD8, CD25, Foxp3, TCR Vα2, and TCR Vβ5. The Foxp3 Transcription Factor Staining Buffer Set and the Annexin V-PE Apoptosis Detection Kit were purchased from eBioscience. Mouse BD Fc Block (Purified rat anti-mouse CD16/CD32) was purchased from BD Pharmingen (Franklin Lakes, NJ). The EasySep™ Mouse SCA1 Positive Selection Kit was purchased from StemCell Technologies Inc. (Cambridge, MA). Chicken Egg Ovalbumin ELISA Kit was purchased from Alpha Diagnostic International Inc. (San Antonio, TX). Rabbit anti-OVA polyclonal antibody (PoAb) was purchased from Abnova (Walnut, CA). HRP-labeled Goat anti-mouse IgG antibody was purchased from Invitrogen (Carlsbad, CA). X-VIVO 10 media was purchased from Lonza Walkersville Inc. (Walkersville, MD). The QIAamp DNA Blood Mini Kit was purchased from QIAGEN (Germantown, MD). GoTaq® Green Master Mix was purchased from Promega (Madison, WI). Ovalbumin (OVA) protein was purchased from Sigma (St. Louis, MO). 3-[(3-cholamidopropyl)dimethylammonio]-1-propanesulfonate (CHAPS, the zwitterionic detergent) was purchased from MP Biomedicals (Solon, OH). Dulbecco's Phosphate-Buffered Saline (DPBS) was purchased from Thermo Fisher Scientific (Carlsbad CA). Nexaband surgical glue was from 3M Animal Care Products (St.Paul, MN).

### Mice

All the animals used in this study were in a C57BL/6 genetic background. WT/CD45.1, WT/CD45.2, OT-II/CD45.2, and CAG-OVA mice used in this study were purchased from the Jackson Laboratory (Bar Harbor, ME) and maintained in our animal facility. Isoflurane or Xylazine/Ketamine was used for anesthesia. All mice were kept in pathogen-free microisolator cages at the animal facilities operated by the Medical College of Wisconsin. Animal studies were performed according to a protocol approved by the Institutional Animal Care and Use Committee of the Medical College of Wisconsin. Animal number in each group was determined by the Resource Equation Method (54–56).

### Vector construction and lentivirus production

The full-length cDNA for chicken ovalbumin was a kind gift from Dr. John Routes (Medical College of Wisconsin, Milwaukee, WI). The lentiviral vector pWPT-2bOVA, harboring the OVA expression cassette under control of the platelet-specific αIIb promoter, was constructed by replacing the FVIII cassette in the pWPT-2bF8 vector (31) with full-length chicken ovalbumin (1.16 kb) cDNA. For the pWPT-2bVpOVA lentiviral vector, a 2.31 kb VWF propeptide cassette with mutated PACE/furin cleavage sites (40) was incorporated into pWPT-2bOVA between the αIIb promoter and the OVA expression cassette. For the pWPT-2bGFP lentiviral vector, the GFP expression cassette from the pWPT-GFP vector was used to replace the FVIII cassette in the pWPT-2bF8 vector (31).

Recombinant lentivirus was generated using a similar protocol as described in our previous reports (31, 57). Briefly, lentivirus were generated from HEK 293T cells cotransfected by calcium phosphate precipitation of 3 plasmids: (1) pWPT-2bOVA, pWPT-2bVpOVA, or pWPT-2bGFP; (2) pCMVΔR8.91; and (3) pMD.G, using the roller bottle system. After 18 h, cell medium was replaced by fresh medium containing 10 mM n-butyric acid (Sigma). Viral supernates were harvested at 48 and 72 h after transfection. The infectious lentivirus was concentrated by centrifugation at 4°C, 6,000 g for 20 h and resuspended in X-VIVO 10 serum-free media. The quality of virus was assessed by methods including (1) viral vector titering by qPCR as described in our previous report (31) using primers specific for sequence located within the LTR region of the vector to determine the number of lentiviral particles and (2) integration titering by qPCR using the same primers amplifying a fragment of the LTR region in DNA purified from a pro-megakaryocytic cell line, Dami cells, that were transduced with lentivirus to determine the infectious titer. Lentivirus was stored frozen at −80°C until utilized.

### HSCs transduction and transplantation

Sca-1^+^ cells from WT/CD45.2 mice or OT-II/CD45.2 TCR transgenic mice were isolated using the EasySep™ Mouse SCA1 Positive Selection Kit following the protocol provided by the manufacturer. Sca-1^+^ cells were transduced with lentivirus following the procedures as described in our previous report (31, 37, 57). Because of the VWF propeptide in the 2bVpOVA construct, the viral titers of 2bVpOVA were 32-fold lower than that of 2bOVA using the same virus production protocol. Thus, we doubled the volume of 2bVpOVA lentivirus to transduce cells compared to the 2bOVA. After transduction, approximately 1x10^6^ cells in 300 μl X-VIVO 10 media were transplanted via retro-orbital venous injection into 6-week-old WT/CD45.1 recipients that were preconditioned with 660 cGy TBI using a cesium irradiator (Gammacell 40 Exactor, Best Theratronics, Ltd., Ottawa, Canada). Age-and sex-matched animals were randomly assigned to the three groups for receiving the transplantation of either 2bOVA-, 2bVpOVA-, or 2bGFP-transduced Sca-1^+^ cells. Animals were analyzed starting at 3 weeks after transplantation. Blood samples were collected monthly by retro-orbital bleeds, and plasma, leukocytes, and platelets were isolated as previously described (36).

### OVA expression analysis

Three assays were used to determine OVA expression in transduced recipients: (1) PCR detection of proviral OVA expression; (2) immunoelectron microscopy of the intracellular location of OVA; and (3) ELISA assay determination of OVA protein expression levels.

#### PCR analysis

Genomic DNA was purified from peripheral leukocytes using the QIAamp DNA Blood Mini Kit and amplified with PCR GoTaq Master Mix. One hundred nanograms of DNA purified from peripheral leukocytes were used to amplify the OVA expression cassette using primers 5′-CTCGAGACGCGTGCCATGGGCTCCATCGGTGCAGCAAGC-3′ and 5′-GTCGACTCAAGGGGAAACACATCTGCC-3′. WT murine FVIII (mFVIII) was used as an internal control to confirm the DNA integrity using primers as we previously reported (37). dH2O was used as a negative control and pWPT-2bOVA plasmid DNA was used as a positive control.

#### Immunogold electron microscopy

EM studies were performed by Clive W. Wells in the Electron Microscopy Facility at the Medical College of Wisconsin. Platelets were isolated and prepared following procedures described in our previous report (35, 39). Platelet pellets were fixed and embedded in Lowicryl K4M resin. Ultrathin sections (60 nm) were collected on formvar/carbon coated copper grids. Sections were incubated with rabbit anti-OVA PoAb and mouse anti-VWF MoAb and then probed with goat anti-rabbit (5 nm) and goat anti-mouse (10 nm) colloidal gold probes, respectively. The sections were observed using a JEM2100 transmission electron microscope (Japanese Electron Optics Ltd, Tokyo, Japan) operating at 80kv. Platelets from an untransduced WT mouse were used as a control. Isotype IgG controls were used in parallel for each staining.

#### OVA ELISA assays

OVA levels in mouse plasmas and platelet lysates or releasates were quantitated by ELISA assay using the Chicken Egg Ovalbumin ELISA Kit following the protocol provided by the manufacturer. For the platelet lysate OVA ELISA assay, 1 × 10^7^ platelets were lysed in 50 μl of 0.5% CHAPS and lysates were serially diluted for assay. For the platelet releasate assay, platelets were stimulated with platelet agonists, which include ADP (Chrono-log Corporation),

and epinephrine (BIO/DATA Corporation) and the murine thrombin receptor activation peptide (GYPGKF-NH2), as described in our previous report (35) and releasates were serially diluted for the assay. For the plasma assay, plasma samples were diluted 1:8 and standards were constructed using diluent containing 1:8 of WT mouse plasma. Samples from WT mice were used as negative controls.

### Flow cytometry analysis

Flow cytometry analysis was used to determine chimerism, T cell populations, and apoptotic CD4 T cell in transduced recipients. One hundred microliters of whole blood were collected from retro-orbital bleeds using 1:10 of 3.8% sodium citrate anticoagulant. Whole blood counts were performed using the **Vet ABC Hematology Analyzer** (Scil Animal Care Company, Gurnee, IL). Red cells were lysed using Gey's solution (0.155 M NH_4_Cl and 0.01 M KHCO_3_). Leukocytes were stained for cell markers CD45.1, CD45.2, CD4, CD8, CD25, Foxp3, Vα2, and Vβ5. For cell surface marker (CD45.1, CD45.2, CD4, CD8, CD25, Vα2, and Vβ5) staining, cells were resuspended in 50 μl of DPBS containing Fc Block (1:50) and incubated for 10 min to block non-specific binding. Cells were then stained with 100 μl of DPBS containing a combination of multiple fluorophore-conjugated antibodies at 4°C for 30 min. For Foxp3 staining, cells were fixed, permeabilized and stained using the Foxp3 Transcription Factor Staining Buffer Set following the protocol provided by the manufacturer.

For lymphoid organ T cell population analysis, animals were sacrificed at 20 weeks after transplantation. Lymphoid organs [spleen (SP), lymph nodes (LN), and thymus (Thy)] were isolated and ground up for single cell preparation. Red cells were lysed with Red Cell Lysing Buffer (Sigma). Cells were washed with DPBS, and 1 × 10^6^ cells were used for each panel to stain the cell markers described above. For apoptotic cell staining, the Annexin V-PE Apoptosis Detection Kit was used following the protocol provided by the manufacturer with the exception of using DAPI in place of 7-AAD. After staining, cells were washed with 1 ml DPBS, resuspended in 200 μl of DPBS buffer containing 1.5 μg/ml DAPI, 2.0% BSA, and 0.02% NaN_3_. All samples were run on a BD LSRII Flow Cytometer (BD Biosciences, Sparks, MD) and analyzed using FlowJo software (FlowJo, LLC, Ashland, OR). Samples from WT/CD45.1 and WT/CD45.2 or OT-II mice were used as controls. Compensation for multicolor staining was performed before each experiment.

### *In vitro* T cell proliferation study

Splenocytes were isolated from transduced recipients and labeled with CellTrace Violet using the CellTrace™ Violet Cell Proliferation Kit (Life Technologies, Carlsbad, CA). Four and a half million cells/well were cultured in flat bottom 96-well plates (BD Falcon, Franklin Lakes, NJ) with 300 μl of completed RPMI-1640 media containing 50 ng/ml OVA (Sigma-Aldrich, St. Louis, MO) for 96 h. Recombinant human factor IX (rhF9) was used as an unrelated antigen control. Concanavalin A [ConA, a mitogen to stimulate non-specific T cell proliferation 58) (Sigma) was used as a positive control for CD4 T cell proliferation. After culture, cells were collected and stained with anti-CD4, anti-CD45.2, and anti-Foxp3 antibodies. Zombie Red^TM^ Fixable Viability Kit (BioLegend) staining was used to exclude dead cells. Cells were analyzed by LSRII flow cytometry (BD Biosciences), and data were analyzed using FlowJo software.

### OVA immune responses study

Sixteen weeks after transplantation, WT/CD45.1 animals that received WT/CD45.2 Sca-1^+^ cells transduced with 2bOVA, 2bVpOVA, or 2bGFP lentivirus were immunized with full-length ovalbumin protein by intraperitoneal injection at a dose of 100 μg/mouse and boosted with 20 μg/mouse 2 weeks later. One week after the 2nd immunization, 100 μl of blood was collected from retro-orbital bleeds and plasma was isolated. Plasmas from recipients before and after OVA immunization were used to determine the titers of anti-OVA total IgG using ELISA assay following the procedures described in our previous report (32).

Some recipients subsequently received skin graft transplantation from CAG-OVA transgenic mice. After applying Xylazine/Ketamine anesthetics and buprenorphine analgesic, full-thickness tail skin grafts from CAG-OVA transgenic mice were transplanted onto a graft bed on the right front side of recipients' backs, and the edges were sealed with surgical glue. Antibiotic ointment was applied on top of the skin graft and secured by a bandage. Seven days after transplantation, bandages were removed, and animals were monitored for skin graft acceptance. The rejection was recorded as the day when the graft was no longer attached to the recipient.

### Statistical analysis

All data are presented as the mean plus or minus SD, and statistical comparisons of experimental groups were evaluated by the unpaired two-tailed student *t*-test. The one-way analysis of variance (ANOVA) was used to determine whether there were statistically significant differences between the means of three or more groups and the Tukey test was used for multiple comparisons. A value of *P* < 0.05 was considered statistically significant.

## Author contributions

XL: designed the study, performed experiments, and analyzed data. JC: performed experiments and analyzed data. JS: performed experiments and made comments to the manuscript. KA: performed experiments and contributed to animal model development. CB: contributed to study design and made comments to the manuscript. SM: contributed to conception of this study and made comments to the manuscript. JH was XL's Ph.D. mentor and provided administrative support for XL. CW: contributed to conception of this study, helped to design research, interpreted data, and edited the manuscript. QS: designed and conducted research, analyzed data, and wrote the manuscript.

### Conflict of interest statement

The authors declare that the research was conducted in the absence of any commercial or financial relationships that could be construed as a potential conflict of interest.
