# Supplementary material for: Platelet Gene Therapy Promotes Targeted Peripheral Tolerance by Clonal Deletion and Induction of Antigen-Specific Regulatory T Cells
Source: Front Immunol. 2018 Sep 6;9:1950. doi: 10.3389/fimmu.2018.01950 (PMC6136275; doi:10.3389/fimmu.2018.01950)
Supplement: Supplementary file 1 [file Data_Sheet_1.PDF]

Figure 1
Supplemental
Figure 1

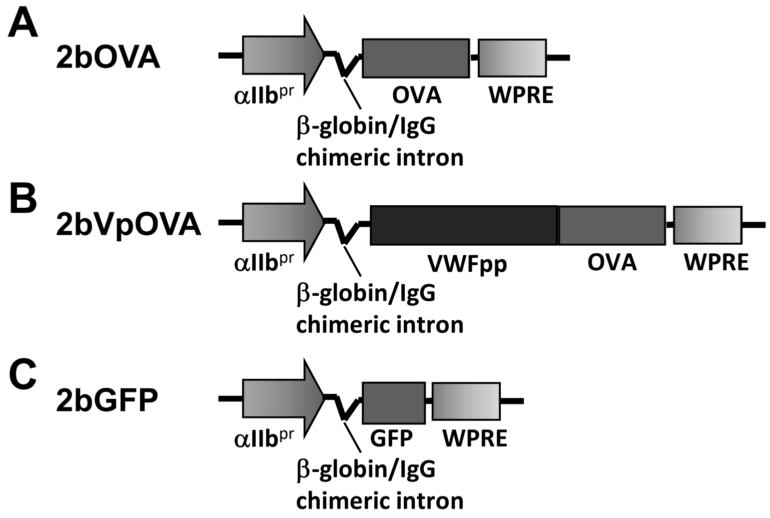

Figure 1 Supplemental Figure 1. Platelet-targeted OVA construct and genetic analysis. (**A**) Schematic diagram of the 2bOVA construct. 2bOVA expression cassette (from 5' to 3'): the human GPIIb gene promoter (αIIb promoter), chimeric intron, full-length chicken ovalbumin (OVA) cDNA, and Woodchuck Hepatitis Virus (WHP) Posttranscriptional Regulatory Element (WPRE). (**B**) Schematic diagram of the 2bVpOVA construct. 2bVpOVA expression cassette (from 5' to 3'): the αIIb promoter, chimeric intron, VWF propeptide (Vp), OVA cDNA, and WPRE. (**C**) Schematic diagram of the 2bGFP construct, which serves as a control vector. 2bGFP expression cassette (from 5' to 3'): the αIIb promoter, chimeric intron, and green fluorescence protein (GFP) cDNA, and WPRE.

Figure 4 Supplemental Figure 1

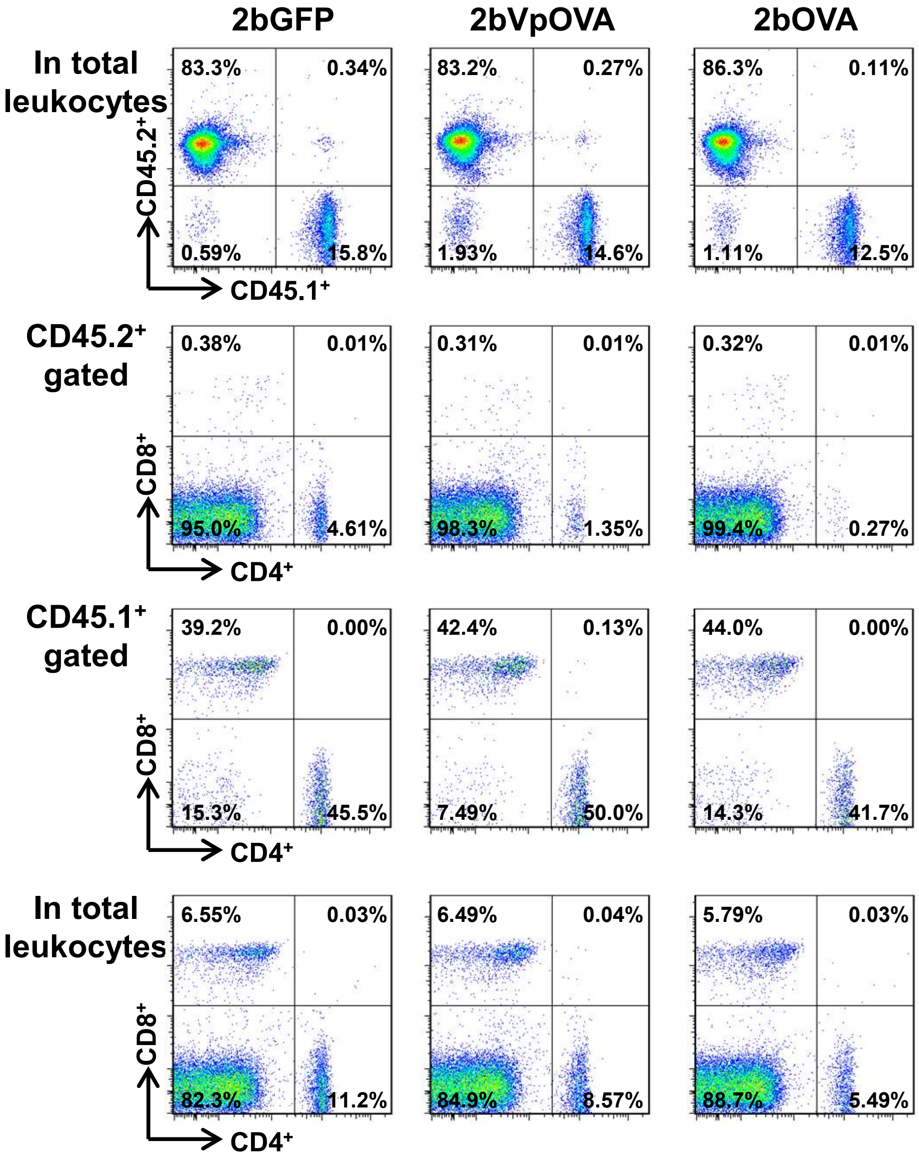

Figure 4 Supplemental Figure 1. Flow cytometry analysis of donor- and recipient-derived T cells in peripheral blood. Leukocytes were isolated from peripheral blood cells and stained with CD45.1, CD45.2, CD4, and CD8. Representative dot plots from the time point of 6 weeks after BMT are shown.

Figure 5 Supplemental Figure 1

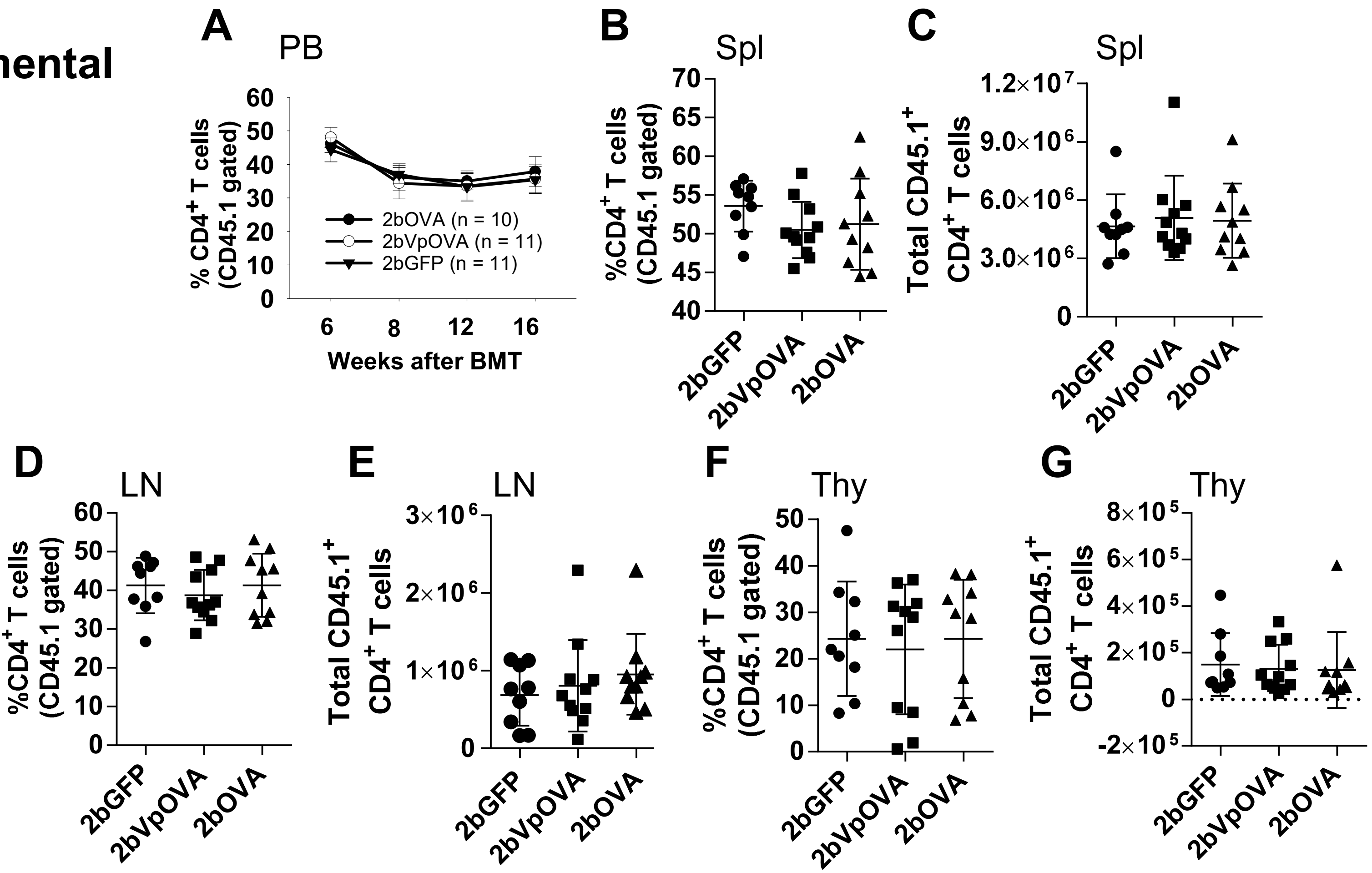

Figure 5 Supplemental Figure 1. Flow cytometry analysis of recipient-derived CD4 T cells in peripheral blood (PB) and lymphoid organs. Blood samples were collected during study course. Five months after BMT, animals were sacrificed. Cells isolated from PB, spleen (Spl), lymph nodes (LN), and thymus (Thy) and stained with CD45.1, CD45.2, CD4, and CD8. Recipient-derived ($CD45.1^+$) cells were gated and analyzed for $CD4^+$ T cells. (**A**) Average percentage of CD4 T cells in PB among recipient residual endogenous leukocytes at each time point. (**B**) Percentage of $CD4^+$ T cells among recipient-derived ($CD45.1^+$) leukocytes in spleen. (**C**) Total number of recipient-derived CD4 T cells in spleen. (**D**) Percentage of $CD4^+$ T cells among recipient-derived leukocytes in lymph nodes. (**E**) Total number of recipient-derived $CD4^+$ T cells in lymph nodes. (**F**) Percentage of single positive CD4 T cells among recipient-derived leukocytes in thymus. (**G**) Total number of recipient-derived single positive CD4 T cells in thymus. Data shown were summarized from two independent experiments. Data were expressed as the mean ± SD. Statistical comparisons of experimental groups were evaluated by the one way ANOVA and there are no statistically significant differences among groups.

Figure 8
Supplemental
Figure 1

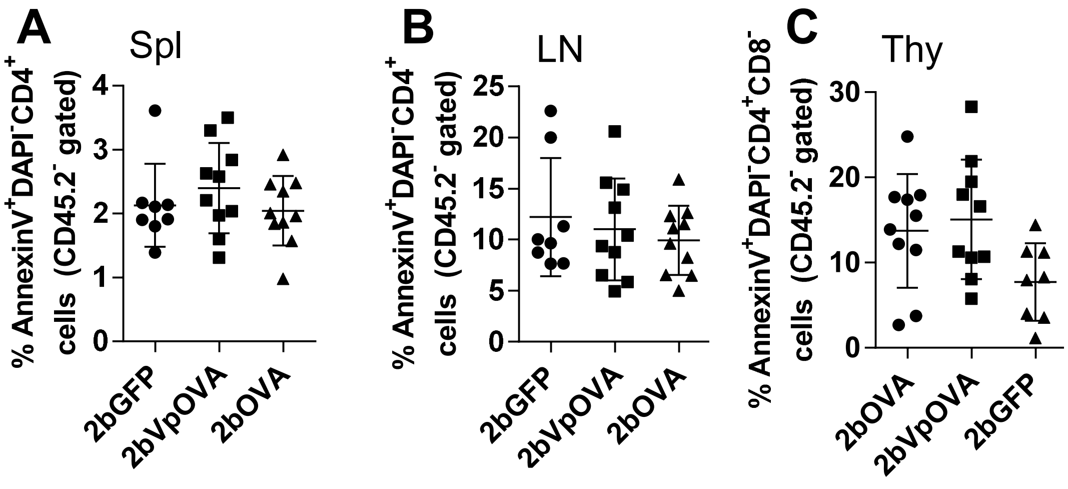

Figure 8 Supplementary Figure 1. Flow cytometry analysis of AnnexinV expression in recipient-derived CD4 T cells in lymphoid organs. Five months after transplantation, animals were sacrificed. One million cells from spleen, lymph nodes, and thymus were stained with CD45.2, CD4, CD8, Annexin-V, and DAPI. Recipient-derived CD4 T cells ($CD4^+CD45.2^-$) were gated and analyzed for Annexin-V^+DAPI^- cells. (**A**) Percentage of apoptotic cells among recipient-derived CD4 T cells in spleens (Spl). (**B**) Percentage of apoptotic cells among recipient-derived CD4 T cells in lymph nodes (LN). (**C**) Percentage of apoptotic cells among recipient-derived single positive CD4 T cells in thymus (Thy). Data shown were summarized from two independent experiments. Data were expressed as the mean ± SD. Statistical comparisons of experimental groups were evaluated by the one way ANOVA and there are no statistically significant differences among groups.

.

Figure 9 Supplemental Figure 1

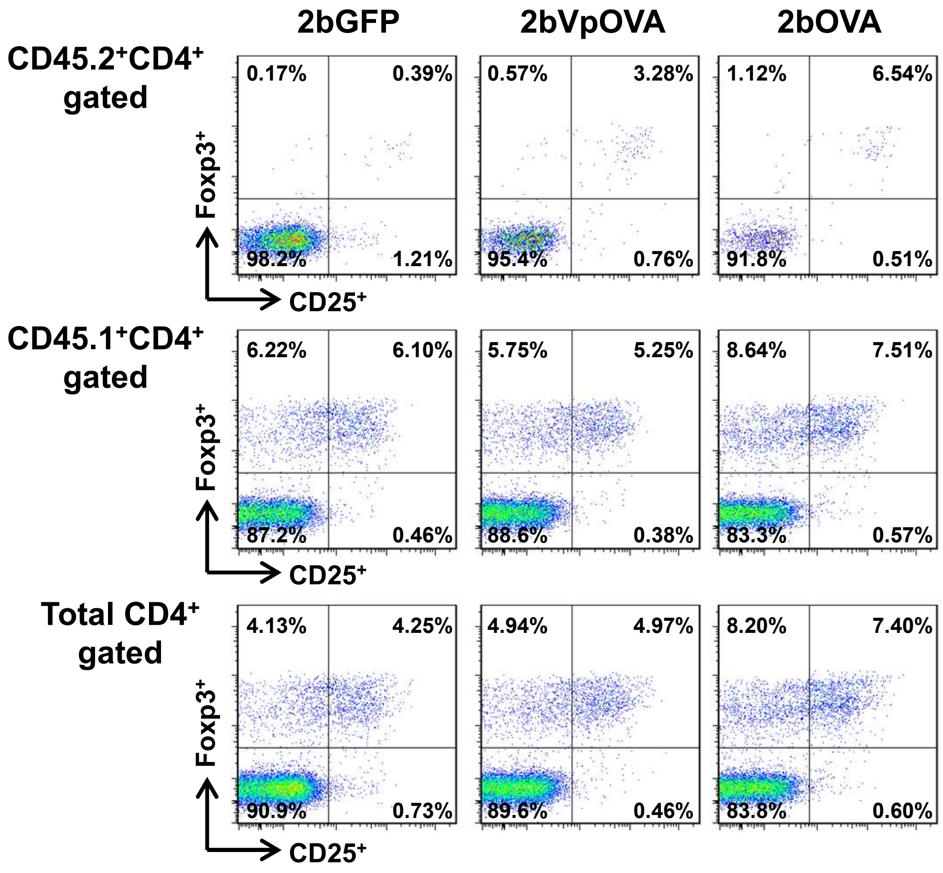

Figure 9 Supplemental Figure 1. Flow cytometry analysis of donor- and recipient-derived Treg cells in peripheral blood. Leukocytes were isolated from peripheral blood and stained with CD45.1, CD45.2, CD4, CD8, CD25, and Foxp3. Representative dot plots from the time point of 4 months after BMT are shown.

Figure 9 Supplemental Figure 2

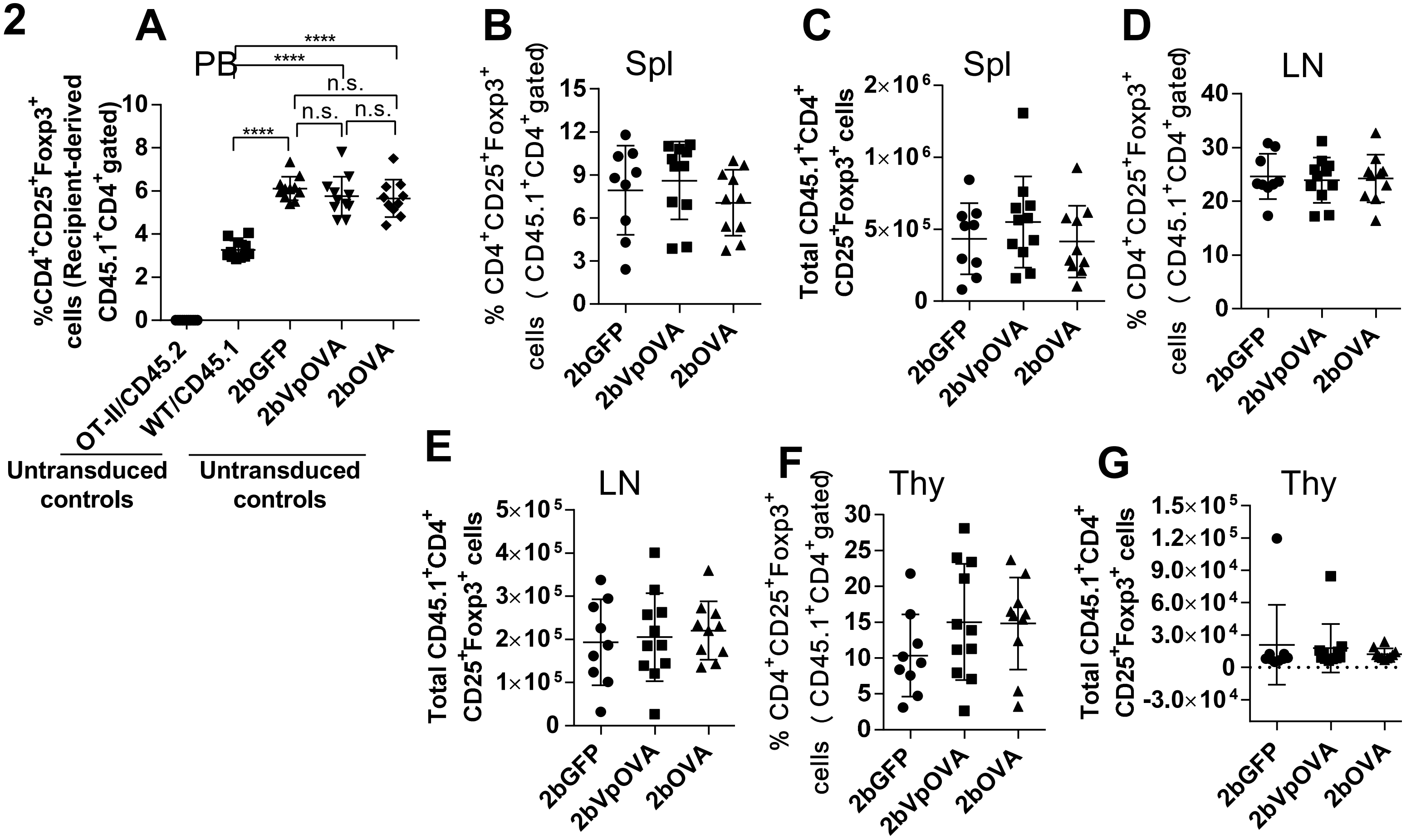

Figure 9 Supplemental Figure 2. Flow cytometry analysis of recipient-derived Treg cells. Leukocytes from peripheral blood (4 months post-BMT) or lymphoid organs (5 months post-BMT) were stained with CD45.1, CD45.2, CD4, CD8, CD25, and Foxp3. Recipient-derived ($CD45.1^+$) cells were gated and analyzed for Treg ($CD4^+CD25^+Foxp3^+$) cells. (**A**) Percentage of Treg cells among-recipient-derived CD4 T cells in peripheral blood (PB). (**B**) Percentage of Treg cells among recipient-derived CD4 T cells in spleen (Spl). (**C**) Total number of recipient-derived Treg cells in spleen. (**D**) Percentage of Treg cells among recipient-derived CD4 T cells in lymph nodes. (**E**) Total number of recipient-derived Treg cells in lymph nodes (LN). (**F**) Percentage of Treg cells among recipient-derived single positive CD4 T cells in thymuse (Thy). (**G**) Total number of recipient-derived Treg cells in thymus. Data shown were summarized from two independent experiments. Data were expressed as the mean ± SD. Statistical comparisons of experimental groups were evaluated by the one way ANOVA and there are no statistically significant differences among groups.
